# Effects of 16 weeks of two different high-protein diets with either resistance or concurrent training on body composition, muscular strength and performance, and markers of liver and kidney function in resistance-trained males

**DOI:** 10.1080/15502783.2023.2236053

**Published:** 2023-07-29

**Authors:** Reza Bagheri, Mehdi Kargarfard, Ramin Sadeghi, David Scott, Donny M Camera

**Affiliations:** a University of Isfahan, Department of Exercise Physiology, Faculty of Sport Sciences, Isfahan, Iran; b Mashhad University of Medical Sciences, Nuclear Medicine Research Center, Mashhad, Iran; c Deakin University, Institute for Physical Activity and Nutrition, School of Exercise and Nutrition Sciences, Geelong, Australia; d Monash University, School of Clinical Sciences at Monash Health, Clayton, Australia; e Swinburne University, Department of Health and Biostatistics, Melbourne, Australia

**Keywords:** Concurrent exercise, protein intake, skeletal muscle adaptation, exercise performance

## Abstract

**Purpose:**

It is unclear whether resistance (RT) and concurrent training (CT; resistance plus endurance training) combined with different protein intakes have differential effects on muscle hypertrophy, strength, and performance. Therefore, we compared the effects of two high-protein diets (1.6 or 3.2 g.kg^−1.^d^−1^) during 16 weeks of either CT or RT alone in resistance-trained males.

**Methods:**

Forty-eight resistance-trained males (age: 26 ± 6 yr, body mass index: 25.6 ± 2.9 kg.m^−2^) performed 16 weeks (four sessions·w^−1^) of CT or RT with either 1.6 g.kg^−1.^d^−1^ protein (CT1; *n* = 12; RT1; *n* = 12) or 3.2 g.kg^−1.^d^−1^ protein (CT2; *n* = 12; RT2; *n* = 12). Training adaptations were assessed pre-, mid-, and post-intervention.

**Results:**

All measures of performance (endurance, vertical jump, and pull-up), lean mass, muscle strength, and power significantly increased post-intervention in all groups, but peak power gains were greater in RT2 compared with RT1 and CT1 (*p* < .05). VO_2max_ significantly increased in both CT groups (*p* < .001). Select biochemical markers of kidney and liver function significantly increased within the RT2 and CT2 groups (*p* < .05), however, no between-group differences were apparent (*p* > .05).

**Conclusions:**

With the exception of peak power, intake of 1.6 g.kg^−1.^d^−1^ of protein appears sufficient to maximize gains in lean mass, muscle strength, performance, and aerobic capacity during both RT and CT without influencing markers of kidney and liver function, indicating this daily protein amount is effective and safely tolerated in young, healthy adults.

## Introduction

Physical performance in sports requires the integration of physiological, biomechanical, metabolic, psychological, nutritional, and training features. Athletic performance capability can be augmented following training programs incorporating resistance training (RT) and endurance training (ET). Resistance training is the most effective training method for improving anabolic-related adaptations (muscular strength, power, or endurance) in trained adults [1]. On the contrary, ET can promote improvements in VO_2max_ as well as increased cardiovascular health and function, and skeletal muscle oxidative capacity [2,3]. Considering training adaptations between ET and RT are largely divergent (and may be influenced by the type and participant’s characteristics), integrating both exercise modalities into a single training program is intuitively necessary to simultaneously maximize anabolic, metabolic, and oxidative adaptation responses. Concurrent training (CT) is generally characterized by the incorporation of RT and ET into the same training program. Previous studies have demonstrated that CT can augment muscle strength, anaerobic power, aerobic capacity, and maximal velocity contraction responses [4–7]. Such adaptations are paramount for athletic populations involved in sports such as basketball, rowing, and rugby, which require anabolic, aerobic, and anaerobic-based adaptations for optimal performance [8,9]. In this respect, and in accordance with the specificity of exercise training principles, exercise modalities in a CT program are likely to rely on the biomechanics and energy systems of a particular athletic activity [10].

Despite these positive adaptation responses with CT, several lines of evidence have reported attenuated increases in muscle strength, power, and hypertrophy with CT compared to RT performed in isolation, known as the “interference effect” [10–15]. Such blunted anabolic training responses are inconsistently reported in the literature and can be dependent upon the training experience of participants, sequence/order of training bouts, and modes of exercise performed [16–20]. Several suggested approaches to circumventing the interference effect have centered on extended recovery periods (i.e. 6–24 hours) between training sessions, performing cycling rather than running as ET, and incorporating post-exercise nutritional strategies [21]. Regarding nutrition, numerous studies have observed enhanced muscular adaptations (i.e. strength, lean mass, or power) with protein ingestion (i.e. diet or supplements) in conjunction with RT [1,22–29]. In contrast, much less consideration has been directed toward the effects of protein intake on exercise adaptation responses with CT. We previously demonstrated similar increases in lean mass and muscle strength in recreationally active males following 12 weeks of RT in isolation (three sessions. w^−1^) and CT (six sessions. w^−1^) combined with a high protein diet (2 g.kg^−1.^d^−1^) [4]. In contrast, select measures of lower body anaerobic power-based adaptations (Wingate) were attenuated with CT compared to RT in isolation, suggesting some training responses are still susceptible to the interference effect with CT and high dietary protein availability. Intriguingly, greater increases in lean mass (3.2 kg vs. 2.2 kg) were recently reported with higher protein intake (~2.2–2.4 g.kg^−1.^d^−1^) compared to ~1.0 g.kg^−1.^d^−1^ after three but not six months of CT in recreationally trained males [30], suggesting the possibility for differences in daily protein availability to modulate adaptation responses with CT. Findings from a recent systematic review provide support for protein supplementation to enhance increases in skeletal muscle mass and strength/power with CT with the post-exercise intake of ~0.49 g/kg bodyweight high-quality protein purported to maximize post-exercise rates of myofibrillar protein synthesis in this context [31].

A systematic review and meta-analysis by Morton and colleagues suggested 1.6 g.kg^−1.^d^−1^ protein was sufficient to maximize fat-free mass (FFM) gains following RT [23]. In contrast, others have speculated that the minimal daily needs of dietary protein in trained individuals should be approximately 2 g.kg^−1.^d^−1^ [29]. Notably, these daily protein suggestions are based on work incorporating RT only. Given greater energetic stresses inherent due to increased training volumes with CT compared to single-mode exercise training, it is possible that required dietary protein amounts are higher for CT [32]. Therefore, to determine whether the potential interference effects of CT on lean mass, strength, and power adaptations can be attenuated, we investigated the effects of two protein diets well above the current recommended daily allowances ([RDAs], 1.6 or 3.2 g.kg^−1^.d^−1^) during 16 weeks of either CT or RT alone. We also sought to determine the effects of these high protein diets on muscular performance, maximal oxygen uptake (VO_2max_), biochemical markers of liver and kidney function, and potential associations between gains in lean mass with all muscular performances (strength, power, endurance, VO_2max_, vertical jump, and pull-up). Based on the currently available evidence, albeit from RT conducted in isolation [23], we hypothesized that a daily protein amount of 1.6 g.kg^−1^.d^−1^ would be sufficient to maximize maximal strength, hypertrophy, performance, and power adaptations with CT compared to both the 3.2 g.kg^−1^.d^−1^ protein condition and RT. We speculate that the 1.6 g.kg^−1^.d^−1^ amount will provide sufficient availability of amino acids for muscle tissue to be utilized for all for required metabolic anabolic cellular processes to maximize adaptation responses despite the potential for RT to be conducted in a compromised energy state due to the concurrent performance of glycogen-depleting, ET-based exercise.

## Methods

### Participants

The present study recruited 48 young, healthy, resistance-trained males aged between 18 and 36 years via advertisements on social media. Interested participants were informed of the study and testing procedures either over the phone or in person at local gyms. By self-report, participants were required to submit a health and fitness history questionnaire, verifying their previous training background of three sessions per week with at least 1 year of RT experience (three to four sessions per week), sleeping for at least 7 to 8 hours during the 24-hour day, not taking any steroids or any illegal agents known to increase muscle size for the previous year, lower than ~1.6 g. kg^−1^.d^−1^ of protein ingestion, and being free from musculoskeletal disorders. Participants deemed eligible according to the criteria mentioned above provided written and verbal consent to participate. In addition, a medical history questionnaire was obtained when consenting, and participants were asked to return to complete the study procedures. The protocol was reviewed by the Institutional Human Subject Committee and the Ethics Committee of the University of Isfahan (IR.UI.REC.1400.098) and carried out in accordance with the Declaration of Helsinki. This study has been registered with the Iranian Registry of Clinical Trials (IRCT20191204045612N2).

### Study design

**Figure 1.**
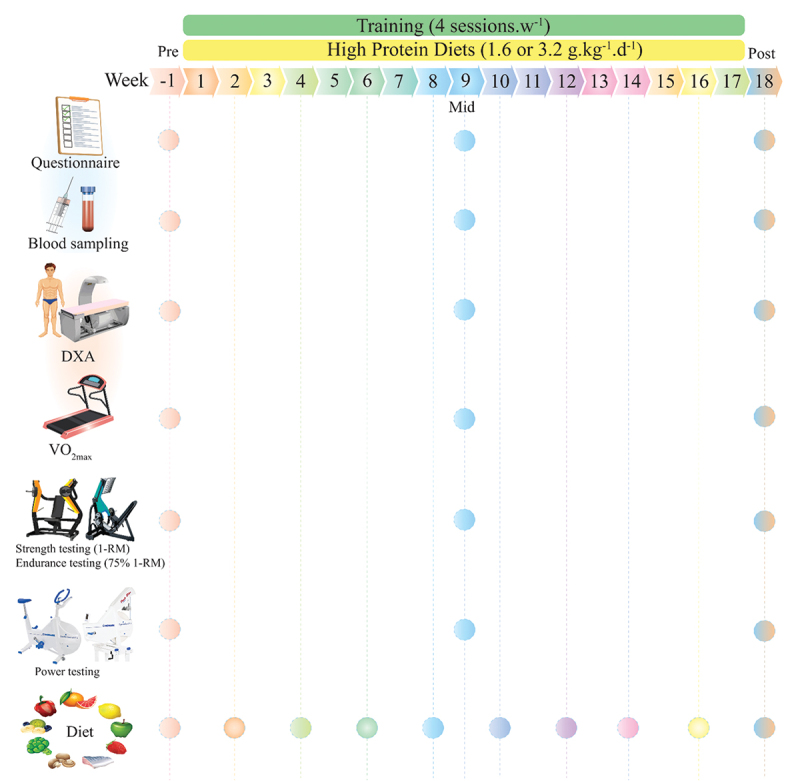
Schematic overview of study timeline.

An overview of the study procedures is shown in Figure 1. After baseline measurements (described subsequently), participants were familiarized with the study tests and procedures and randomly assigned to one of four groups using an online resource (www.randomizer.org): CT +1.6 g.kg^−1^.d^−1^ of protein (CT1; *n* = 12), CT +3.2 g.kg^−1^.d^−1^ of protein (CT2; *n* = 12), RT +1.6 g.kg^−1^.d^−1^ of protein (RT1; *n* = 12) or RT +3.2 g.kg^−1^.d^−1^ of protein (RT2; *n* = 12). Our original intent regarding the length of this study was six months; however, due to the Coronavirus Disease 2019 (COVID-19) pandemic, we voluntarily decided to end the study at 16 weeks. Therefore, measurements were collected at baseline, week 9, and week 18 (i.e. two weeks post-training intervention) during the same time of day (−1 hour). Participants first completed four preliminary testing days: on the first visit, questionnaires were assessed; on the second visit, blood draw and body composition were performed; on the third visit, VO_2max_ and performance tests were performed; on the fourth visit, participants completed chest and leg press one-repetition maximum (1-RM), muscular endurance tests (75% 1-RM), and muscular power. After performing these tests, participants met the study dietitian for an introductory consultation to discuss food preferences as well as target protein and energy intakes prior to the initiation of training programs. To assess sleep quality and health status, the Pittsburgh Sleep Quality Index (PSQI) and the General Health Questionnaire-28 (GHQ-28) were used, respectively [33]. All mentioned procedures were performed in exactly the order for all time measurements.

### Anthropometry and body composition

Participants were asked to report to the laboratory hydrated after an overnight fast, with a 24-hour dietary recall collected before testing. Participants were instructed to void completely within 30 minutes of the test to minimize hydration status errors and advised to refrain from caffeinated beverages, alcohol, and other diuretics 12 hours prior to measurements. Participants’ body mass and height were measured with a digital scale (Lumbar, China) to the nearest 0.1 kg and a stadiometer (Race industrialization, China) to the nearest 0.1 cm, respectively. Total lean mass, body fat percentage (BFP), and estimated visceral adipose tissue (VAT) were assessed using whole-body dual-energy x-ray absorptiometry (DXA; Hologic, Discovery, Wi [S/N 93,045 M]). Briefly, participants were asked to lay supine on the DXA examination table wearing shorts for approximately 7 minutes while a low dose of radiation scanned their entire body. For DXA measurements, previous test re-test reliability in our laboratory is as follows: BFP intraclass correlation coefficient (ICC) = 0.998; coefficient of variation (CV): <1%; lean mass: ICC = 1.00; CV: <1%. All DXA scans were conducted by the same technician, analyzed with the image compare mode for serial examination software feature (Hologic APEX software, version 4.5.3.2), and followed strict manufacturer guidelines for calibration and testing procedures as per the formerly published study [34].

### Maximal strength testing

Maximal strength was determined using 1-RM for plate-loaded leg press and chest press. This testing (1-RM) also was performed to determine training intensity for RT protocols. Before the beginning of the test, the researchers explained the purpose, attendant risks, discomforts, responsibilities of the participant, benefits, inquiries, and freedom of consent. Participants were instructed to refrain from alcohol for 48 hours, caffeinated drinks for 12 hours, and food intake for 2 hours before the testing session; however, water consumption was allowed. Participants performed a general 10 min warm-up (5 min slow running on a treadmill; 3–5 km speed, or elliptical; with 5–10 level) and specific warm-up activities (5 min, e.g. medicine ball twist 1 × 10, medicine ball wood chops 1 × 10, straddled toe touch 2 × 5, dynamic quadriceps stretch 1 × 5, medicine ball squat 1 × 5–8) before the test. The participants then performed two attempts, recording their highest lifted weight and number of repetitions. The number of repetitions to fatigue did not exceed 10. Participants were allowed 3 to 5 minutes rest periods between attempts, and there was no arousing stimulus during testing. After the testing session, participant’s maximal strength was predicted using the formula: 1-RM= weight/(1.0278–0.0278×reps) [21]. Chest and leg press exercises were used as upper and lower body strength measures, and 1-RM was used to determine individualized RT prescription.

### Muscular endurance

The participants rested for 5 minutes after the 1-RM testing prior to completing the muscular endurance test in the morning (9:00–10 a.m.). Participants were instructed to perform leg- and chest press exercises at 75% of the 1-RM to test muscular endurance, denoted as the number of successful repetitions completed prior to technical failure [34].

### Training volume

RT volume was calculated using the following formula in each session and was reported weekly [35]:

1. Resistance training volume = [repetitions (n) × sets (n) × load or selected weight (kg)].
2. Endurance training volume was calculated using the following formula: Total ET volume: [work + rest].
3. Work: [Intensity × maximum aerobic power (MAP) × (set × duration [as noted in training protocol] × 0.06)].
4. Rest: [Intensity × MAP × (set × duration [as noted in training protocol] × 0.06)].
5. Intensity: percent of MAP; Set: number of repetitions of each session; Duration: spent time (minutes); 0.06: Convert watts to kilojoules

## Muscular power

Upper- and lower-body anaerobic power was assessed via Monark Wingate cycle ergometry (Monark model 894e, Vansbro, Sweden) as previously described [4,36]. Briefly, participants were acquainted with the test and instructed to stay seated in the saddle for the test duration. Participants cycled or cranked against a pre-determined resistance (7.5% of body mass for the lower body test and 5.5% for the upper body test) as fast as possible for 30 seconds. Participants were verbally encouraged to pedal as hard and fast as possible throughout the whole 30 seconds test. Peak power output was documented in real-time during the test using Monark Anaerobic test software (3.3.0.0).

## Performance testing

Maximal vertical jump height and total pull-ups (1 set) were assessed. Each participant generally performed the following warm-up: a 5-min run/bike on a treadmill or cycle ergometer at a self-directed leisurely pace followed by a dynamic warm-up consisting of 10 yards each of high knees, butt kicks, side shuffles, and karaoke running drill, and finally ten pushups and ten bodyweight squats. Participants then rested for 2–3 min prior to commencing the performance tests. Subsequently, the following tests were performed in the order given: vertical jump – highest value with a maximum number of three attempts; pull-ups – highest repetitions with a maximum number of three attempts. For both tests, there was a rest interval of approximately 60–180 seconds.

## VO_2max_ testing

Briefly, the participants performed an Ekblom-Bak (EB) test with a constant pedal frequency of 60 revolutions per min (rpm). The protocol of the EB test is described in detail elsewhere [37,38]. The test started with 4 min of cycling at the standard work rate with a resistance of 0.5 kiloponds (kp), ~ 30 watts (W). The second submaximal work rate was individually chosen to obtain a rating of perceived exertion (RPE) of ~14 and the test was terminated if the participant rated a perceived exertion higher than 16 [37]. The selection of the higher work rate was based on the participants’ self-reported physical status and training habits. HR and VO_2_ were recorded as the mean at the last minute for each work rate. The estimated VO_2max_ was calculated using the sex-specific EB prediction equations, previously shown to have good validity and reliability in mixed populations [38,39]. The test was conducted on a Monark cycle ergometer model 828E (Monark Exercise AB, Vansbro, Sweden).

### Blood tests

Fasting blood samples (5 ml) were taken from the cubital vein using standard procedures following an 8-hour overnight fast at the same time of day (8:00–9:00 a.m.) in pre, mid, and post-testing. Blood samples were centrifuged at 1000 g at 4°C for 15 min, with aliquots of serum frozen in liquid N2 and stored at − 80°C before analysis. Liver enzymes (alanine transaminase [ALT; intra-assay CV: 1.81%; inter-assay CV: 2%]), aspartate aminotransferase [AST; intra-assay CV: 2.01%; inter-assay CV: 2.54%], and gamma-glutamyl transferase [GGT; intra-assay CV: 1.56%; inter-assay CV: 0.92%], creatinine [intra-assay CV: 1.60%; inter-assay CV: 2.24%], and Urea Nitrogen (BUN) [intra-assay CV: 2.20%; inter-assay CV: 3.36%] were measured in serum. Liver and kidney function markers were measured in duplicates using Pars Azmoon kits and the spectrophotometric method (DiaSys Diagnostic Systems GmbH, Germany). These were performed after 48 hours of rest.

### Resistance training

Participants in the two RT groups performed four sessions/week (Saturday, Monday, Wednesday, and Thursday) involving a supervised, linear periodized training program comprising two upper and two lower-body sessions each week. Prior to each RT session, participants performed 10 minutes of general (5 min slow running on a treadmill; 3–5 km speed, or elliptical; with 5–10 level) and specific warm-up activities (5 min, e.g. medicine ball twist 1 × 10, medicine ball wood chops 1 × 10, straddled toe touch 2 × 5, dynamic quadriceps stretch 1 × 5, medicine ball squat 1 × 5–8). Participants then completed an upper-body RT program consisting of seven exercises (chest press, lateral pulldown, standing barbell shoulder press, standing shoulder shrugs, bicep curl, triceps press down, and abdominal crunch) 2×/wk and six exercises of lower-body RT program (45-degree leg press, back squats, seated leg curl, Barbell hip thrusts, back extension, and calf raises) performed for 2×/wk. Participants performed three sets of 12 repetitions with 75% of 1-RM for weeks 1–4, 3 sets of 10 repetitions with 80% of 1-RM for weeks 5–8, 4 sets of 8 repetitions with 85% of 1-RM for weeks 9–12, and 4 sets of 6 repetitions with 90% of 1-RM for weeks 13–16. Rest intervals between exercises and sets lasted no longer than 2 minutes [40]. The periodized RT program was based on our previous work [40] and following recommendations by the National Strength and Conditioning Association [41]. Participants were provided verbal encouragement and feedback during and after each set. Training data for each participant were logged, permitting us to guarantee that training effort was maximized within each training session and that participants successfully implemented progressive overload in an individualized fashion. In addition, study personnel supervised all training throughout the study. A detailed outline can be found in Table 1.

**Table 1.** Resistance training program.

Week	Intensity (1-repetition maximum [RM])	Set	Repetition	Rest (seconds)
1	75%	3	12	45
2	75%	3	12	45
3	75%	3	12	45
4	75%	3	12	45
5	80%	3	10	60
6	80%	3	10	60
7	80%	3	10	60
8	80%	3	10	60
9	No training - Testing session	----	----	----
10	85%	4	8	90
11	85%	4	8	90
12	85%	4	8	90
13	85%	4	8	90
14	90%	4	6	120
15	90%	4	6	120
16	90%	4	6	120
17	90%	4	6	120
18	No training - Testing session	----	----	----

### Concurrent training

Participants in the two CT groups performed four sessions/week (Saturday, Monday, Wednesday, and Thursday) comprising RT at the beginning of the session, followed by ET as an exercise order sequence recommended [42] to minimize possible interferences in muscle anabolism. Prior to each CT session, participants performed 10 minutes of general (5 min slow running on a treadmill; 3–5 km speed, or elliptical; with 5–10 level) and specific warm-up activities (5 min, e.g. medicine ball twist 1 × 10, medicine ball wood chops 1 × 10, straddled toe touch 2 × 5, dynamic quadriceps stretch 1 × 5, medicine ball squat 1 × 5–8). Participants then undertook the same RT program as described above. Immediately following the completion of RT, participants then performed endurance cycle training on ergometers that consisted of a mixture of hill simulation rides of varying intensities (25–110 of MAP), moderate-intensity continuous training at 50% MAP, moderate-intensity interval training (MICT) at 70% MAP, and high-intensity interval training (HIIT) at 100% MAP. Moderate-intensity intervals were separated by a 60-s recovery period at ~ 40% MAP to establish a 2.5:1 or 5:1 work-to-rest ratio. High-intensity intervals were separated by 20- to 60-s recovery periods, completed at ~ 40% MAP, to establish a 1:5, 1:2, or 1:1 work-to-rest ratio. All cycling sessions were preceded by 3–5 min of cycling at ≤50 W. Progressive overload was applied by manipulating the number of intervals and relative intensity of load throughout. A detailed outline can be found in Table 2(a, b).

**Table 2.** A. Overview of 16-week endurance training program. B. the detailed endurance training program.

A					
Session Outlines					
Session Descriptor	Work Interval	Rest Interval	Work Power % MAP	Rest Power % MAP	Repeats
Hill Stimulation	7 min	----	40	----	—–
	5 min	----	52.5	----	----
	3 min	----	62.5	----	----
	3 min	----	70	----	----
	5 min	----	25	----	----
	10 Sec	----	40	----	----
	10 Sec	----	50	----	----
	10 Sec	----	60	----	----
	10 Sec	----	70	----	----
	10 Sec	----	80	----	----
	10 Sec	----	90	----	----
	10 Sec	----	100	----	----
	5 min	----	25–40	----	----
6 × 2.5	2.5 min	1 min	70%	40%	6
7 × 2.5	2.5 min	1 min	70%	40%	7
4 × 4	4 min	1 min	70%	40%	4
6 × 4	4 min	1 min	70%	40%	6
7 × 4	4 min	1 min	70%	40%	7
8 × 4	4 min	1 min	70%	40%	8
HIIT Session A	10 sec	50 sec	100%	40%	6
	60 sec	60 sec	100%	40%	1
	20 sec	40 sec	100%	40%	3
	----	4 min	----	40%	----
Repeat all parts 2x after 4 min rest					
HIIT Session B	3 min	1 min	70%	40%	3
	10 sec	50 sec	100%	40%	6
	60 sec	60 sec	100%	40%	1
	20 sec	40 sec	100%	40%	3
	-	4 min	—–	40%	----
Repeat all parts 2x after 4 min rest					
Steady State	30 min	----	50%	----	----
B					
	Session				
Week	1	2	3	4	
1	Hill Simulation	6 × 2.5	Hill Simulation	4 × 4	
2	7 × 2.5	Hill Simulation	6 × 2.5	8 × 4	
3	4 × 4	Hill Simulation	7 × 2.5	Hill Simulation	
4	6 × 4	Hill Simulation +5%	6 × 2.5	4 × 4	
5	6 × 2.5	Hill Simulation +10%	7 × 4	Hill Simulation	
6	Hill Simulation + 15%	Steady State	Hill stimulation	7 × 4	
7	Hill Simulation	8 × 4	Hill Simulation	HIIT A	
8	7 × 4	Hill Simulation	HIIT A	HIIT B	
9	No training - Testing session	----	----	----	
10	8 × 4	Hill Simulation	HIIT A	Steady State	
11	Hill Simulation	Steady State	Hill Simulation +5%	8 × 4	
12	HIIT B	Hill Simulation +10%	HIIT B	6 × 4	
13	HIIT B	Hill Simulation +10%	HIIT B	7 × 2.5	
14	8 × 4	Steady State	HIIT A	Hill Simulation +10%	
15	Hill Stimulation + 15 %	HIIT A	7 × 2.5	HIIT A	
16	HIIT A	Hill Stimulation + 15 %	4 × 4	HIIT B	
17	HIIT A	Steady State	7 × 4	6 × 4	
18	No training - Testing session	----	----	----	

Abbreviation: MAP, maximum aerobic power; HIIT, high-intensity interval training.

### Diet

Participants completed six 24 h dietary logs (4 nonconsecutive weekdays and 2 nonconsecutive weekend days) to determine habitual protein intakes. To assist in achieving their targeted protein intake (i.e. 1.6 or 3.2 g.kg^−1.^d^−1^), participants consumed a 40 g of isolated whey protein (Wisser nutrition, Iran) beverage upon cessation of every training session that comprised the following nutrition profile per scoop (28 g): calories,110; total fat, 0.5 g; saturated and trans-fat, sugars, and dietary fiber, 0 g; sodium, 50 mg; potassium, 112 mg; total carbohydrate, 2 g; protein, 24 g. Other remaining protein quantities were consumed via foods, and habitual dietary protein intake remained stable throughout the intervention for all groups.

Our rationale for implementing the 1.6 g.kg^−1.^d^−1^ protein group was based on the previously mentioned work by Morton et al. (2018), where this daily amount was recommended to maximize FFM gains following RT [23]. As no study has currently investigated the effects of protein availability above 2–2.2 g.kg^−1.^d^−1^ on training adaptation responses with CT, we sought to ensure there was a clear difference in the amount of protein intake between groups. Thus, we chose to double the 1.6 g.kg^−1.^d^−1^ amount for the comparison high protein group (i.e. 3.2 g.kg^−1.^d^−1^) while also ensuring this amount can be safely tolerated. In support, previous work by Antonio and colleagues demonstrated this amount (~2.51–3.32 g.kg^−1.^d^−1^) to exert no harmful effects on liver and kidney function markers [43].

Participants attended consultations with an accredited practicing dietitian every two weeks, where they were provided guidelines to reach protein and energy needs, including the distribution of protein intake throughout the day across 4–7 meals with 20–40 g of protein per meal to maximize muscle protein synthesis (MPS) [44,45]. Macronutrient composition was supervised during the study, with total energy intake (TEI) and protein intake a focus. Carbohydrate and fat intake were suggested to be within the Acceptable Macronutrient Distribution Range for these macronutrients (45–65% and 20–35% TEI for carbohydrate and fat, respectively). Participants were asked to remain in a positive energy balance to alleviate any potential of energetic stress-related interferences to anabolic adaptations [46,47]. Food records were kept daily by participants throughout the study using mobile phone applications Easy Diet Diary (Xyris Software Pty Ltd, AUS, for those with iPhones, Apple Inc, USA; *n* = 18) and My Fitness Pal (MyFitnessPal Inc., USA) for those with Android-based devices; *n* = 26). All dietary intake data were analyzed using (Diet Analysis Plus, version 10; Cengage) to ensure the same food database was used for all analyses.

## Statistical analysis

The sample size was calculated using PASS.15 software in which an *F* test, repeated measures, and within-between interaction ANOVA revealed that 40 participants were needed to detect a medium effect (Cohen’s f = 0.25) with a significance level of α = 0.05 and 80% power for changes in muscular strength (the primary outcome of this study) post-exercise training intervention [4]. We recruited 20% more participants (*n* = 8, 2 for each group) due to potential dropouts. The normality of the distribution of all variables was evaluated before performing statistical analyses using the Shapiro – Wilk test; there were no missing values at any time point. Baseline characteristics (at PRE) between groups were reported using mean (SD). Effects of training and nutritional interventions on dependent variables were analyzed using a three × four analysis of variance (ANOVA) with repeated measures (time [pretest vs. mid-test vs. posttest] × group [CT1 vs. CT2 vs. RT1 vs. RT2]) to determine the differences between the treatments over time. When the group-by-time interaction was significant, we used Generalized Estimation Equation (GEE) analysis to determine between-group differences. Analysis of covariance (ANCOVA) was used to assess the changes between pretest and during-intervention nutrient intakes. Cohen’s d effect size (ES) was calculated as post‐training mean minus pre‐training mean/pooled pre‐training standard deviation means [48]. All analyses were performed using R (version 4.1.2), and figure production was performed using GraphPad Prism (version 8.4.3) and R software (version 4.1.2).

## Results

### Participant characteristics

**Figure 2.**
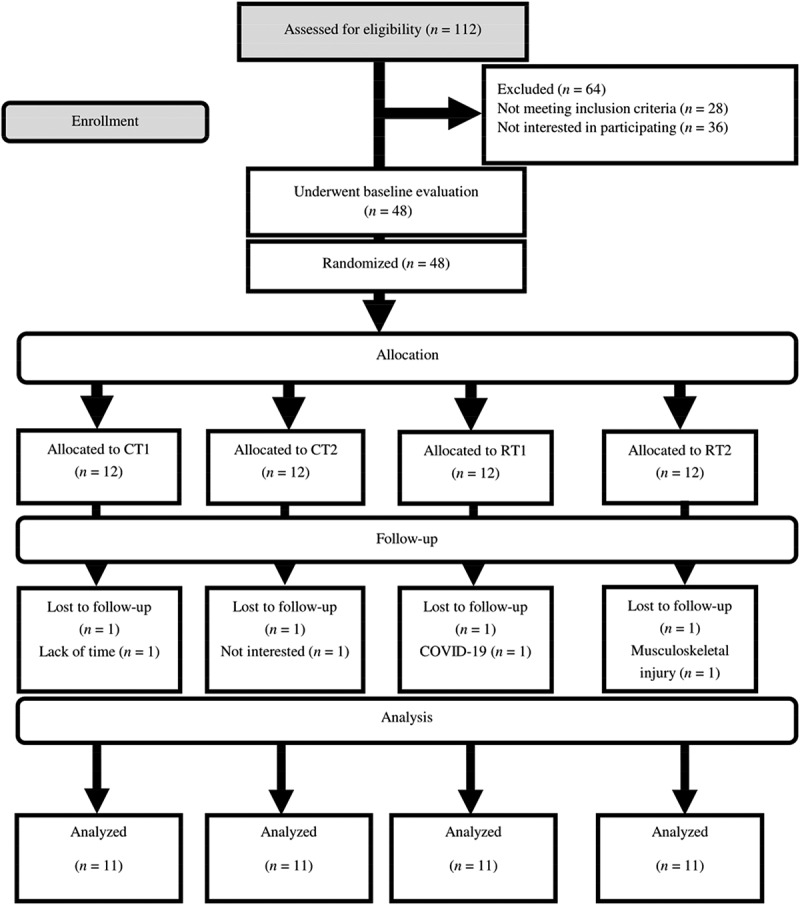
The flow of participant recruitment.

One hundred and twelve participants were assessed for eligibility. Twenty-eight did not meet the inclusion criteria, while 36 were not interested in participating after the first interview (Figure 2). One participant from each group (due to lack of time, not being interested, COVID-19, or musculoskeletal injury) withdrew from the study. There were no significant between-group differences in all baseline characteristics (Table 3). There were no differences between groups for PSQI (*p* = 0.923), GHQ-28q (*p* = 0.421), or training experience (*p* = 0.475).

**Table 3.** Baseline characteristics of the participants.

	CT1	CT2	RT1	RT2	p-value
**Measure**					
**Anthropometry, training experience, health and sleep questionnaires**					
Age (y)	27 ± 6	25 ± 7	26 ± 6	28 ± 5	0.875
Height (cm)	178 ± 5	179 ± 8	180 ± 7	182 ± 6	0.700
Body mass (kg)	83.8 ± 10.6	81.6 ± 10.7	82.1 ± 9.1	85.2 ± 10.9	0.844
BMI (kg.m^−2^)	26.3 ± 3.4	25.2 ± 3.1	25.1 ± 2.3	25.7 ± 2.9	0.786
Training experience (yr)	3.7 ± 2.2	4.6 ± 2.6	3.5 ± 1.7	4.8 ± 2.4	0.475
PSQI	3 ± 1.1	3.1 ± 1.3	3.2 ± 0.9	3.2 ± 1.1	0.923
GHQ-28	21.9 ± 8.1	19.7 ± 4.8	24.5 ± 9.2	20.1 ± 6.6	0.475
**Body composition**					
Lean mass (kg)	60.5 ± 6	59.7 ± 6.2	59.9 ± 6.6	62.7 ± 9.2	0.748
EST. VAT mass (g)	376.1 ± 221.5	323.7 ± 139.1	330.6 ± 131	313.6 ± 101.4	0.789
BFP (%)	21.8 ± 5.7	21.4 ± 4.1	21.6 ± 3.4	21 ± 4	0.983
**Performance**					
Absolute chest press strength (kg)	96.7 ± 21.5	100.8 ± 19.3	98.2 ± 17.7	107.8 ± 16.4	0.532
Relative chest press strength (kg. kg BM^−1^)	1.16 ± 0.24	1.25 ± 0.32	1.22 ± 0.31	1.28 ± 0.22	0.765
Chest press endurance (r)	11.8 ± 2.4	13.4 ± 1.6	11 ± 2.3	12 ± 2	0.070
Absolute leg press strength (kg)	412.5 ± 76.7	388.2 ± 73.5	390.3 ± 70.8	408.7 ± 69.5	0.810
Relative leg press strength (kg. kg BM^−1^)	4.97 ± 1.12	4.85 ± 1.23	4.83 ± 1.17	4.87 ± 1.08	0.992
Lower body endurance (r)	14.1 ± 2.8	15.3 ± 3.3	14.8 ± 3.1	15.2 ± 2.2	0.769
Absolute upper body power (w)	496 ± 62.4	505 ± 92.6	456.9 ± 61.3	509 ± 70.9	0.258
Relative upper body power (watt. kg BM^−1^)	5.63 ± 0.80	6.32 ± 1.66	5.63 ± 1.07	6.05 ± 1.10	0.470
Absolute lower body power (w)	696.2 ± 91.8	738.8 ± 83	695.8 ± 53	752.1 ± 68.8	0.199
Relative lower body power (watt. kg BM^−1^)	8.38 ± 1.36	9.21 ± 1.81	8.60 ± 1.40	8.96 ± 1.44	0.585
Vertical jump (cm)	50.6 ± 4.5	47.8 ± 8.2	43 ± 6.9	44.9 ± 7.1	0.064
Pull-up (r)	11.3 ± 2.8	13.2 ± 3	12.7 ± 2	14.3 ± 4.3	0.183
VO_2max_ (ml^−1^.kg^−1^.min^−1^)	36 ± 4.5	37.1 ± 6.4	33.9 ± 5.4	36.4 ± 6.9	0.603
**Biochemical markers**					
GGT (u/l)	30.1 ± 5.4	29.2 ± 6.2	30.8 ± 4.7	31.1 ± 6.7	0.888
AST (u/l)	29.6 ± 7.6	27.1 ± 5.7	25.7 ± 6.8	28.8 ± 8.1	0.583
ALT (u/l)	24.1 ± 5.9	27.9 ± 6.6	26.1 ± 6.5	29.1 ± 7.7	0.327
Urea (mg/dl)	17.9 ± 5.2	16 ± 6	17 ± 4.1	18 ± 5.3	0.787
Creatinine (mg/dl)	1.11 ± 0.18	1.06 ± 0.15	1.18 ± 0.23	1.15 ± 0.18	0.512

Values are presented as mean ± standard deviation. Abbreviations: PSQI, Pittsburgh Sleep Quality Index; GHQ-28, General Health Questionnaire; BMI, body mass index; EST. VAT, Estimated visceral adipose tissue; BFP, body fat percentage; VO2max, maximum rate of oxygen consumption; GGT, gamma-glutamyl transferase; AST, aspartate aminotransferase; ALT, alanine aminotransferase; y, year; cm, centimeter; kg, kilogram; kg.m−2, kilogram-meter −2; g, gram; %, percentage; cm2, centimeter 2; g/cm2, gram/centimeter 2; r, repetition; w, watt; ml−1.kg−1.min-1, milliliter−1, kilogram−1.minute−1; pg/ml, picograms/milliliter; ng/ml, nanogram/milliliter; u/l, unit/liter; mg/dl, Milligrams/deciliter; CT1, concurrent training +1.6 g.kg−1.d−1; CT2, concurrent training +3.2 g.kg−1.d−1; RT1, resistance training +1.6 g.kg−1.d−1; RT2, resistance training +3.2 g.kg−1.d−1.

### Body composition

**Figure 3.**
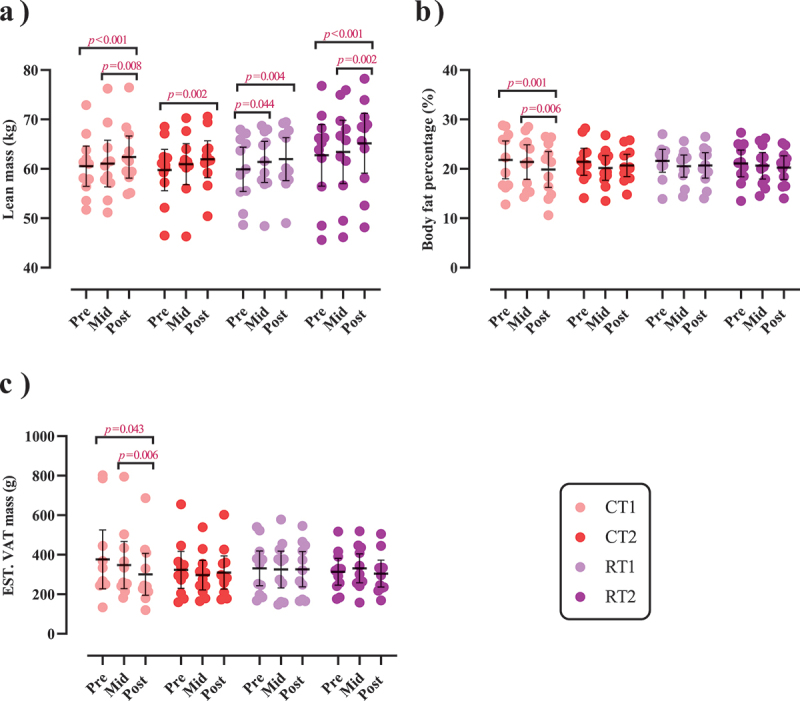
Effects of resistance or concurrent training in combination with high protein diets on body composition. a) lean mass (kg), b) body fat percentage (BFP), and c) estimated visceral adipose tissue (EST.VAT mass [g]). *n*=11 per group, error bars represent 95% confidence interval (CI), and p-values above time points indicate paired sample t-test results. CT1, concurrent training + 1.6 g.kg^−1^.d^−1^; CT2, concurrent training + 3.2 g.kg^−1^.d^−1^; RT1, resistance training + 1.6 g.kg^−1^.d^−1^; RT2, resistance training + 3.2 g.kg^−1^.d^−1^.

Changes in body composition throughout the intervention are shown in Figure 3 and Supplementary Tables S4A and B. There was a significant main effect of time for body mass (*p* < 0.001), BMI (*p* < .001), lean mass [(*p* < .001), (Figure 3a)], BFP [(*p* = .001), (Figure 3b)], and VAT [(*p* = 0.022), (Figure 3c)]. Body mass and BMI significantly increased from pre to post by 2.2% in RT1 (*p* = 0.026) and 2.4% in RT2 (*p* = .022). Lean mass significantly increased from pre to post by 3% in CT1 (*p* < .001), 3.8% in CT2 (*p* = .002), 3.5% in RT1 (*p* = .004), and 4% in RT2 (*p* < .001). BFP significantly decreased from pre to post by 9% in CT1 (*p* = .001). VAT significantly decreased from pre to post by 16.4% in CT1 (*p* = .043).

### Performance

**Figure 4.**
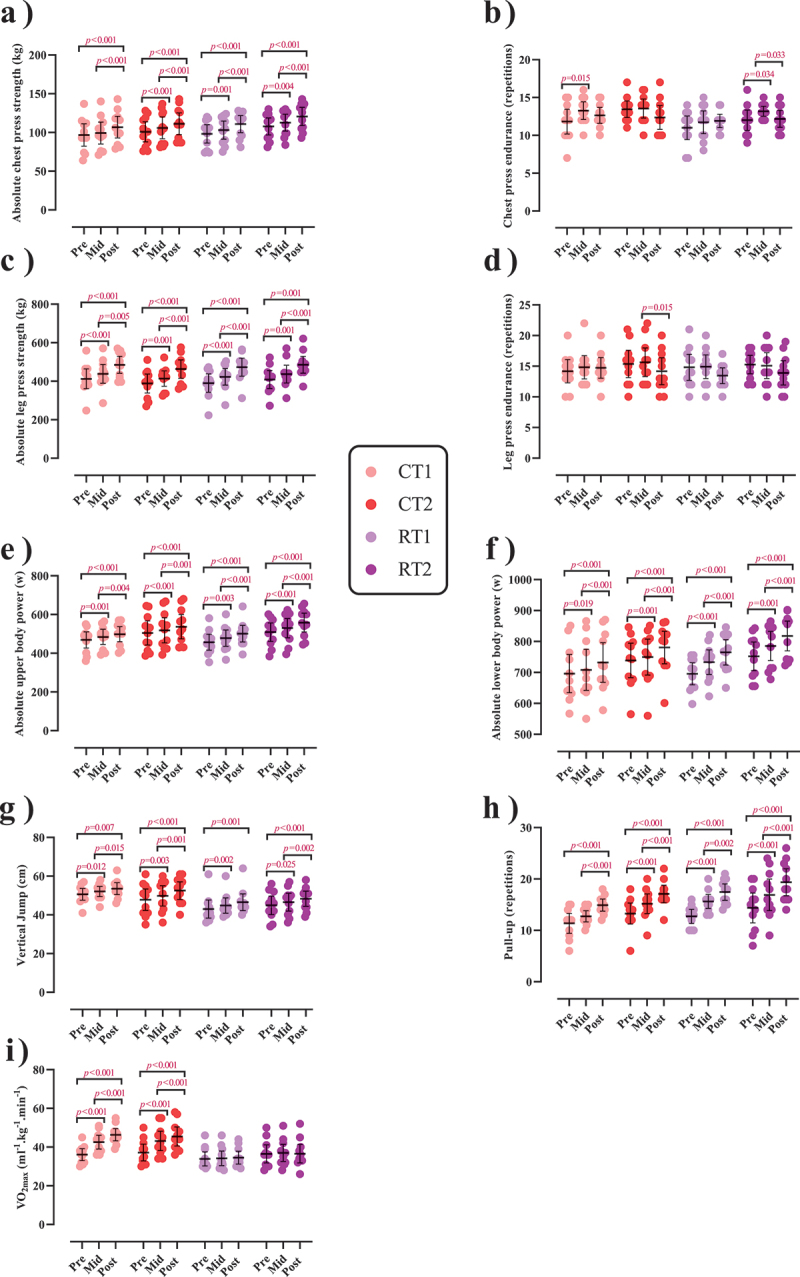
Effects of resistance or concurrent training in combination with high protein diets on muscular performance. a) Absolute chest press strength (kg), b) Chest press endurance (r), c) Absolute leg press strength (kg), d) Leg press endurance (r), e) Absolute upper body power (w), f) Absolute lower body power (w), g) Vertical jump (cm), h) Pull-up (r), and I) VO_2max_ (ml^−1^.kg^−1^.min^−1^). *n* = 11 per group, error bars represent 95% confidence interval (CI), and p-values above time points indicate paired sample t-test results. CT1, concurrent training + 1.6 g.kg^−1^.d^−1^; CT2, concurrent training + 3.2 g.kg^−1^.d^−1^; RT1, resistance training + 1.6 g.kg^−1^.d^−1^; RT2, resistance training + 3.2 g.kg^−1^.d^−1^.

**Figure 5.**
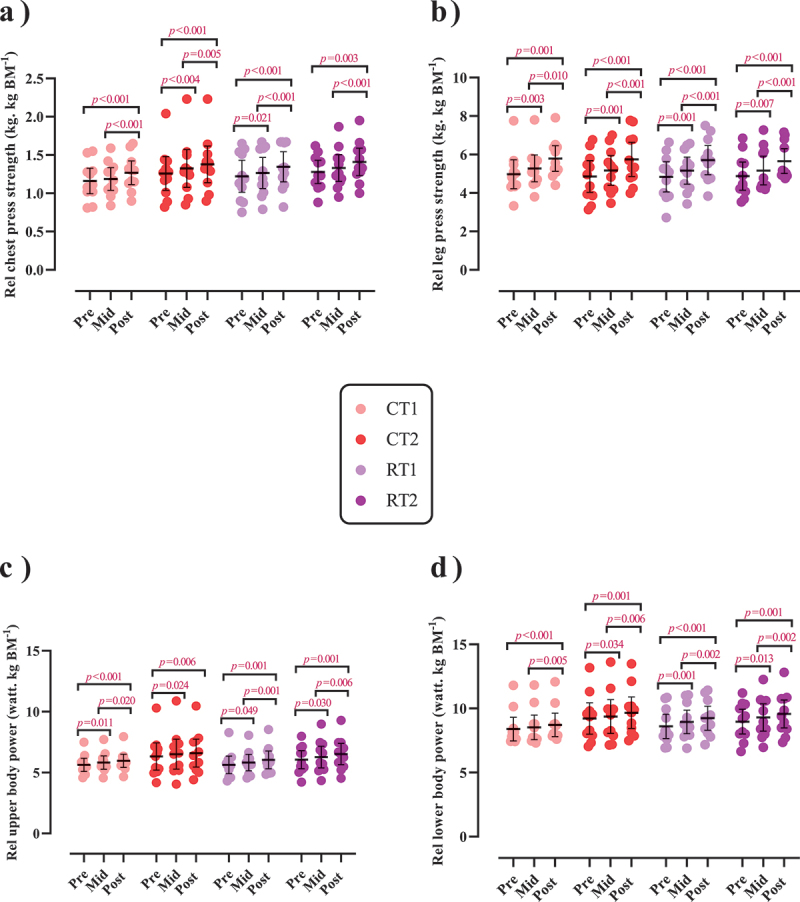
Effects of resistance or concurrent training in combination with high protein diets on relative upper and lower body strength and power. a) Relative chest press strength (kg. kg BM^−1^), b) Relative leg press strength (kg. kg BM^−1^), c) Relative upper body power (watt. kg BM^−1^), and d) Relative lower body power (watt. kg BM^−1^). *n*=11 per group, error bars represent 95% confidence interval (CI), and p-values above each time points indicate paired sample t-test results. CT1, concurrent training + 1.6 g.kg^−1^.d^−1^; CT2, concurrent training + 3.2 g.kg^−1^.d^−1^; RT1, resistance training + 1.6 g.kg^−1^.d^−1^; RT2, resistance training + 3.2 g.kg^−1^.d^−1^.

Changes in performance throughout the intervention are shown in Figures 4 and 5 as well as Supplementary Tables S5A and B.

#### Muscular strength

There was a significant main effect of time for both absolute [(*p* < .001), (Figure 4a)] and relative chest press strength [(*p* < .001), Figure 5a)] as well as for both absolute (*p* < .001; Figure 4c) and relative leg press strength (*p* < .001; Figure 5b). Absolute chest press strength significantly increased from pre to post by 11.2% in CT1 (*p* < .001), 10.4% in CT2 (*p* < .001), 13.4% in RT1 (*p* < .001), and 12.1% in RT2 (*p* < .001). Relative chest press strength significantly increased from pre to post by 10% in CT1 (*p* < .001), 9.5% in CT2 (*p* < .001), 11% in RT1 (*p* < .001), and 10.1% in RT2 (*p* = .003). Absolute leg press strength significantly increased from pre to post by 19.6% in CT1 (*p* < .001), 20.3% in CT2 (*p* < .001), 22.3% in RT1 (*p* < .001), and 20% in RT2 (*p* = .001). Relative leg press strength significantly increased from pre to post by 18.3% in CT1 (*p* < .001), 19.3% in CT2 (*p* < .001), 19.7% in RT1 (*p* < .001), and 17.7% in RT2 (*p* < .001). No between-group differences were observed.

#### Muscular endurance

There was a significant main effect of time for chest press [(*p* = .020), (Figure 4b)], and leg press endurance (*p* = .022; Figure 4d). Chest press endurance significantly increased from pre to mid by 14.9% in CT1 (*p* = .015) and 11.8% in RT2 (*p* = 0.034) while significantly decreasing from mid to post by 7.6% in RT2 (*p* = .033). Leg press endurance significantly decreased from mid to post by 8.9% in CT2 (*p* = .015). No between-group differences were observed.

#### Muscular power

There was a significant group-by-time interaction for absolute upper-body power (*p* = .009; Figure 4e)] and absolute lower-body peak power [(*p* < .001; Figure 4f)]. The increase of absolute lower body power in RT2 was significantly greater (0.1%) than in RT1 (*p* = .044). This was also observed for absolute lower body power (*p* = .033) and compared to CT1 [3.6%, (*p* = .034)]. Regarding relative values, there was a significant main effect of time for both relative upper and lower body power (*p* < .001; Figure 5(c, d), respectively). Relative upper body power significantly increased from pre to post by 5.9% in CT1, 4.4% in CT2, 7.5% in RT1, and 7.4% in RT2 [(*p* < .01; Figure 5C)]. Relative lower body power significantly increased from pre to post by 4.5% in CT1, 3.7% in CT2, 7.6% in RT1, and 6.3% in RT2 [(*p* < .01; Figure 5d)].

#### Muscular performance

There was a significant main effect of time for vertical jump (*p* < .001; Figure 4g) and pull-up (*p* < .001; Figure 4h)]. Vertical jump significantly increased from pre to post by 5.9% in CT1 (*p* = .007), 10.7% in CT2 (*p* < .001), 8.7% in RT1 (*p* = .001), and 8.3% in RT2 (*p* < .001). Pull-up significantly increased from pre to post by 38% in CT1 (*p* < .001), 33.1% in CT2 (*p* < .001), 38.4% in RT1 (*p* < 0.001), and 41.6% in RT2 (*p* < .001). There was a significant group-by-time interaction for VO_2max_ [(*p* < .001), (Figure 4i)]. The increase in VO_2max_ in CT1 was significantly greater (26.94%) than in RT1 (*p* < .001) and RT2 (28.69%, *p* = .045). In addition, the increase in CT2 was significantly greater (20.46%) than in RT1 (*p* = .004).

### Biochemical markers

**Figure 6.**
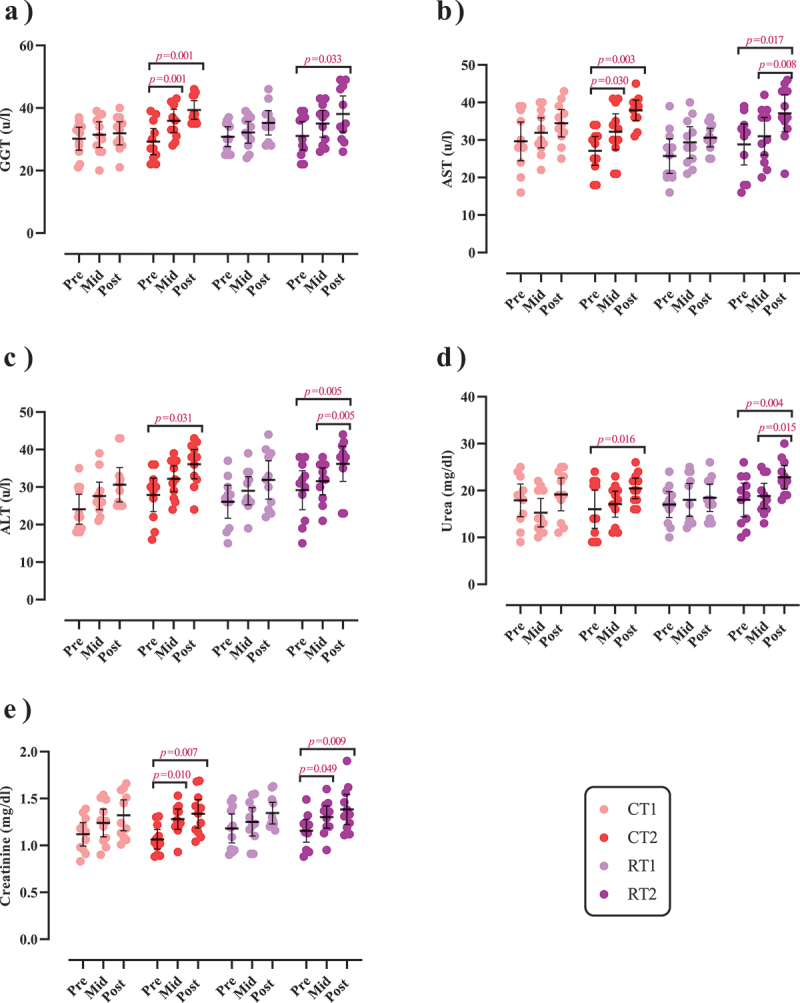
Effects of resistance or concurrent training in combination with high protein diets on markers of liver and kidney function. a) gamma-glutamyl transferase (GGT [(u/l)]), b) Aspartate transaminase (AST [(u/l)]), c) Alanine transaminase (ALT [(u/l)]), d) Urea (mg/dl), and e) Creatinine (mg/dl). *n*=11 per group, error bars represent 95% confidence interval (CI), and p-values above each time points indicate paired sample t-test results. CT1, concurrent training + 1.6 g.kg^−1^.d^−1^; CT2, concurrent training + 3.2 g.kg^−1^.d^−1^; RT1, resistance training + 1.6 g.kg^−1^.d^−1^; RT2, resistance training + 3.2 g.kg^−1^.d^−1^.

Changes in biochemical markers throughout the intervention are shown in Figure 6 and Supplementary Tables S6A and B.

#### Liver function

There was a significant main effect of time for GGT (*p* < .001; Figure 6d), AST (*p* < .001; Figure 6e), and ALT (*p* < .001; Figure 6f). GGT significantly increased from pre to post by 39.7% in CT2 (*p* = .001) and 26.8% in RT2 (*p* = .033). AST significantly increased from pre to post by 49.2% in CT2 (*p* = .003) and 39.4% in RT2 (*p* = .017). ALT significantly increased from pre to post by 41.1% in CT2 (*p* = .031) and 30.6% in RT2 (*p* = .005). There were no changes for any marker in CT1 and RT1 over time (*p* > .05).

#### Kidney function

There was a significant main effect of time for urea (*p* = 0.003; Figure 6g) and creatinine (*p* < .001), Figure 6H). Urea significantly increased from pre to post by 44.3% in CT2 (*p* = .016) and 35.5% in RT2 (*p* = .004). Creatinine significantly increased from pre to post by 28% in CT2 (*p* = .007) and 21.1% in RT2 (*p* = .009). There were no changes for any marker in CT1 and RT1 over time (*p* > .05).

## Dietary assessments

Average dietary intakes at baseline and throughout the intervention are presented in Table 4. There was no significant difference between groups at baseline for any average daily nutrient and energy intake (*p* > .05). Energy intake increased by 20% in CT2 (*p* < .001) and 19% in RT2 from baseline (*p* = .023). ANCOVA indicated that energy intake in CT1 (123.49 ± 14.50 kJ.kg^−1^.d^−1^) was significantly lower than in CT2 (144.37 ± 22.41 kJ.kg^−1^.d^−1^; *p* = .003) and RT2 (137.36 ± 15.39 kJ.kg^−1^.d^−1^; *p* = .041). In addition, energy intake in CT2 (144.37 ± 22.41 kJ.kg^−1^.d^−1^) was significantly greater than in RT1 (124.69 ± 12.36 kJ.kg^−1^.d^−1^; *p* = .002). Protein intake increased from baseline by 84.6% in CT1 (*p* = .001), 216.1% in CT2 (*p* < .001), 45.3% in RT1 (*p* = .005), and 206.6% in RT2 (*p* < .001). Protein intake in CT1 (1.62 ± 0.022 g.kg^−1.^d^−1^) was significantly lower than in CT2 (3.29 ± 0.100 g.kg^−1.^d^−1^) and RT2 (3.25 ± 0.064 g.kg^−1.^d^−1^; *p* < .001) while protein intake in RT1 (1.63 ± 0.031 g.kg^−1.^d^−1^) was significantly lower than in RT2 (3.25 ± 0.064 g.kg^−1.^d^−1^; *p* < .001)). Fat and carbohydrate intake did not significantly change from baseline in any group (*p* > .05).

**Table 4.** Average dietary intake at baseline and throughout the 16-week training intervention.

	Time	
	Baseline	Training
Energy (kJ. kg^−1^.d^−1^)		
CT1	119.9 ± 22.8	123.4 ± 14.5
CT2	122 ± 25	144.3 ± 22.4^a^
RT1	122.8 ± 16.1	124.6 ± 12.3
RT2	118.3 ± 22.3	137.3 ± 15.3^a^
Protein (g.kg^−1.^d^−1^)		
CT1	1.03 ± 0.42	1.62 ± 0.02^a^
CT2	1.16 ± 0.36	3.29 ± 0.10^a^
RT1	1.25 ± 0.37	1.63 ± 0.03^a^
RT2	1.14 ± 0.28	3.25 ± 0.06^a^
Carbohydrate (g.kg^−1.^d^−1^)		
CT1	4.31 ± 0.99	4.05 ± 0.89
CT2	4.42 ± 1.51	4 ± 1.1
RT1	4.43 ± 0.95	4.28 ± 0.76
RT2	3.98 ± 1.23	3.36 ± 0.73
Fat (g.kg^−1.^d^−1^)		
CT1	0.80 ± 0.24	0.74 ± 0.20
CT2	0.75 ± 0.20	0.58 ± 0.22
RT1	0.73 ± 0.24	0.67 ± 0.16
RT2	0.86 ± 0.21	0.70 ± 0.25

^a^p<.05 different from baseline. Abbreviations: CT1, concurrent training + 1.6 g.kg^−1.^d^−1^; CT2, concurrent training + 3.2 g.kg^−1.^d^−1^; RT1, resistance training + 1.6 g.kg^−1.^d^−1^; RT2, resistance training + 3.2 g.kg^−1.^d^−1^.

## Training volume

**Figure 7.**
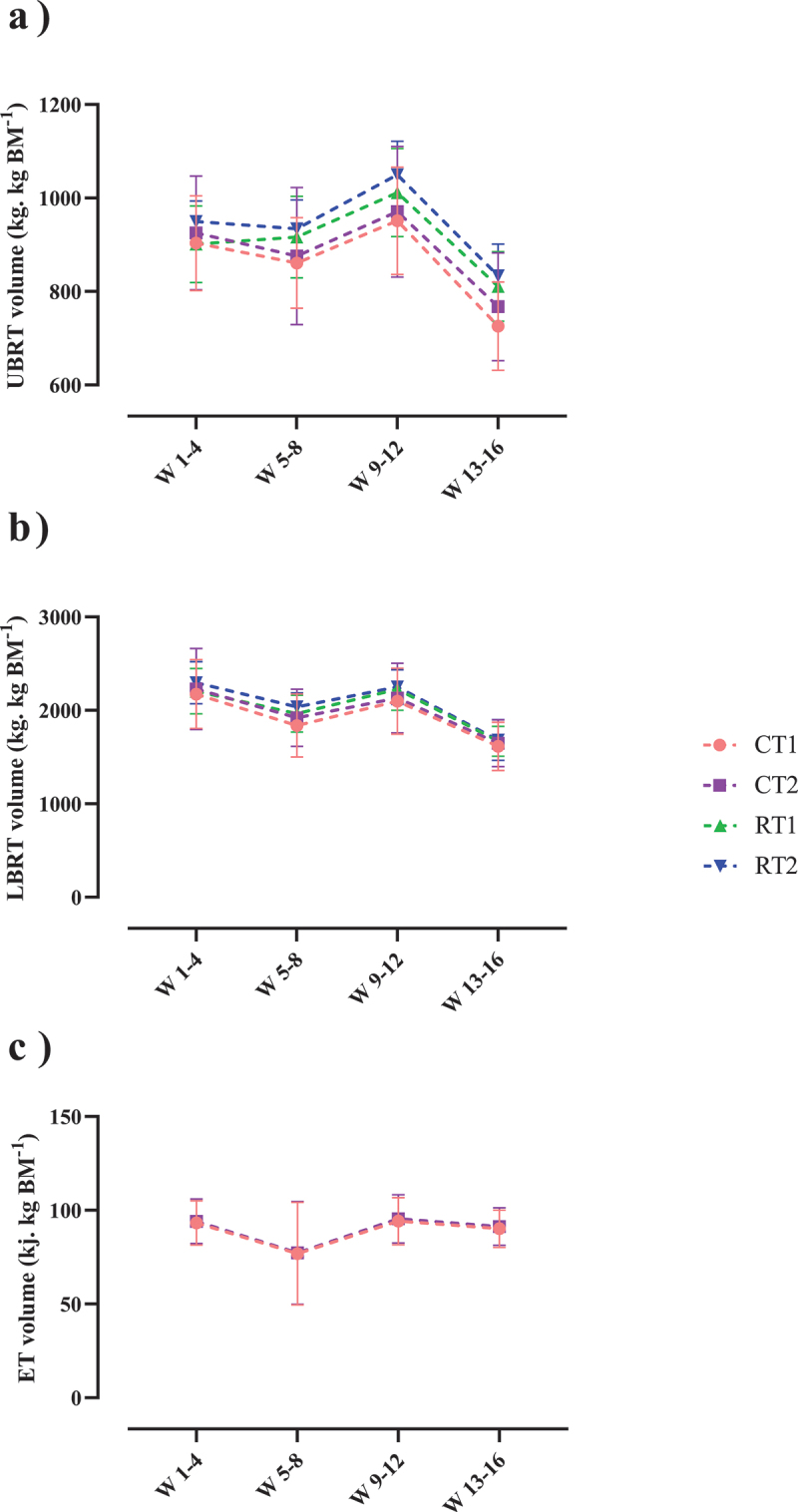
Effects of resistance or concurrent training in combination with high protein diets on training volume. a) Upper body resistance training volume (UBRT volume [kg.Kg BM ^−1^]), b) Lower body resistance training volume (LBRT volume [kg.Kg BM ^−1^]), and c) Endurance training volume (ET volume [kj. Kg BM^−1^]). CT1, concurrent training + 1.6 g.kg^−1^.d^−1^; CT2, concurrent training + 3.2 g.kg^−1^.d^−1^; RT1, resistance training + 1.6 g.kg^−1^.d^−1^; RT2, resistance training + 3.2 g.kg^−1^.d^−1^.

Changes in training volume throughout the intervention are shown in Figure 7. There was a significant main effect of time for upper body and lower body RT volume (*p* < .001; Figure 7(a, b), respectively). Upper body RT volume significantly decreased from weeks 1–4 to weeks 13–16 by 19.6% in CT1 (*p* < .001), 15.4% in CT2 (*p* < .001), 10.6% in RT1 (*p* < .001), and 12.3% in RT2 (*p* = .001). Moreover, lower body RT volume significantly decreased from weeks 1–4 to weeks 13–16 by 25.3% in CT1 (*p* < .001), 24.8% in CT2 (*p* < .001), 26.1% in RT1 (*p* < .001), and 25.2% in RT2 (*p* < .001). However, there was no significant main effect of time for ET volume in either CT1 or CT2 groups (*p* = .245; Figure 7c).

## Discussion

We report that CT, when performed 4 d.wk^−1^ on alternate days combined with two different high protein diets, does not compromise gains in muscular strength, absolute upper body power, or lean mass compared to RT alone combined with high protein diets. Furthermore, only CT induced significant increases in VO_2max_ post-intervention. In contrast, there was attenuation in absolute lower body power post-intervention in CT1 when compared to RT2 but not RT1. Overall, these observations present novel information regarding nutrition practices for maximizing muscle lean mass, strength, performance, and power adaptations with CT.

## Muscle strength and power adaptations

The first major finding from our current work was that gains in strength and absolute upper body power increased similarly in all exercise training and dietary protein groups, indicating that these adaptation responses were not impaired with CT compared to RT. In contrast, increases in absolute lower body power with RT2 were significantly greater than CT1. In his classical work in the 1980s, Hickson showed ET to attenuate maximal strength development in a CT program [49], although subsequent studies with a considerably lower total training volume have reported no interference in maximal strength with CT [14,50,51]. A systematic review and meta‑analysis of 27 studies in 2021 found that CT exclusively impairs lower-body maximal strength development in trained, but not moderately trained or untrained, individuals [52]. Moreover, this attenuation in strength for trained individuals was more pronounced when CT sessions were undertaken within the same session compared to different/separate sessions with an extended recovery between exercise bouts [52]. In contrast, an updated systematic review and meta‑analysis reported no interference in the development of maximal strength with CT compared to strength training in isolation, independent of individual training history/status [19]. Participants in our study possessed previous RT experience (i.e. performing three sessions per week for at least one year). Such training experience/status has been theorized to increase the likelihood of interference in strength development compared to untrained cohorts due to a lower potential for strength adaptations with CT, meaning even a small interference effect would then be sufficient to reduce strength development adaptation in trained individuals similar to those in our study [11]. Nonetheless, and perhaps more confounding in our study design, was that the individual exercise sessions comprising our CT program were undertaken immediately after each other with no recovery period. Both acute and accumulated fatigue induced by ET can decrease the volume or intensity of RT undertaken [53,54], particularly if the ET component of CT involves self-regulated high-intensity ET. Robineau and colleagues previously reported that increases in maximal strength for bench press and half squat were significantly reduced when strength and ET were performed within the same session (i.e. one session immediately after the other) compared to either a 6- or 24-h recovery interval between training sessions in a trained cohort [55]. Interestingly, RT sessions were always performed before ET in that study, which was the same exercise order as in our current work. In addition to acknowledged differences in participant characteristics, exercise training variables (i.e. sets, repetitions, exercise duration), and training length (7 versus 16 weeks) between our current study and work by Robineau et al. [55], another key difference was the high dietary protein component of our study. We speculate the high protein availability with both CT groups in our study may have played a role in the similar maximum strength responses between CT and RT through the established capacity for dietary protein to support and augment skeletal muscle remodeling during post-exercise recovery [56]. In support, a systematic review, meta-analysis, and meta-regression by Morton and coworkers demonstrated protein supplementation (1.6 g.kg^−1.^d^−1^) to enhance RT-induced increases in 1-RM strength in trained (and untrained) individuals [23]. Additionally, within a CT setting, higher protein availability (~2 g.kg^−1.^d^−1^) has been shown to promote greater increases in upper-body 1-RM strength (i.e. bench press) compared to lower protein intake (~1 g.kg^−1.^d^−1^) over 8 [57] and 12 [58] weeks. However, not all studies have reported further beneficial gains in maximum strength following CT with increased protein intake [7,59]. Furthermore, there was no comparison in strength adaptations between CT and an RT-only condition in work by Robineau and colleagues, precluding the possibility that gains in muscle strength may still have been similar (i.e. no interference) between CT with no recovery period between sessions and RT performed in isolation. Considering the limited number of studies into this area, future studies investigating the capacity for different amounts of daily protein intake to augment maximum strength adaptations with CT involving different recovery lengths between RT and ET are required to provide more insight in this area.

In contrast to changes in maximum strength, increases in absolute lower body peak power were significantly reduced with CT1 compared to RT2 as measured by Wingate cycle ergometry. While this attenuation in absolute lower body peak power was not apparent with the CT2 group, we have previously reported blunted responses in relative Wingate peak power output with CT compared to RT only in conjunction with a diet providing 2 g.kg^−1.^d^−1^ of protein [4]. The capacity for CT to compromise explosive muscular power but not maximum strength adaptations (as observed in our past and current work) is in agreement with findings reported by Schumann and colleagues from their recent systematic review and meta‑analysis [19]. Subgroup analysis from this work further showed this reduced development in explosive strength/power with CT to be further compounded if RT and ET modes were performed within the same session compared to if separated by at least three hours of recovery. The basis for this is theorized to result from residual fatigue induced by the ET component of CT affecting neural input to the motor neurons innervating working leg muscle groups before force generation, thus reducing rapid force output [19]. Altered pennation angle and fascicle length adaptations [60], muscle shortening velocity [61], and muscle fiber type changes [62] have also been implicated in dampened muscle power adaptations with CT compared to RT only. Based on previous reviews suggesting more beneficial lower strength adaptations [63,64], RT was performed before the ET component of the CT program in our current study. In theory, this sequential order reduces the likelihood of residual fatigue from ET impairing muscle power adaptation responses with the RT component of a CT session. However, the work by Schumann and colleagues indicates that attenuation in muscle power responses with CT is more related to the close proximity of exercise sessions compared to the exercise order *per se* [19].

## Body compositional changes

Post-intervention gains in lean mass were similar between CT and RT groups regardless of daily dietary protein intake. These results support our earlier work over 12 weeks, where similar increases in total and leg lean mass were observed post-intervention between CT and RT groups with a high protein diet of 2 g.kg^−1.^d^−1^ [4]. Previous studies have observed significantly higher increases in FFM or lean mass following CT with protein intakes of ~ 2.1–2.3 g.kg^−1.^d^−1^ compared to ~1.1 g.kg^−1.^d^−1^ [30,65,66]. These findings collectively indicate a beneficial effect of increased protein intake with CT compared to levels marginally above the current RDA. In further support, a recent systematic review investigating the effects of dietary protein supplementation on longer-term changes in muscle mass with CT identified five of a total of nine studies to enhance post-intervention changes in either lean or FFM following CT with increased protein availability [31]. However, a limitation of many of these studies was that there was no comparison to an RT-only group to ascertain whether these increases in lean mass with CT were of a similar or different (i.e. greater or lower) magnitude. Moreover, the daily protein intakes of these studies were no higher than ~2.2 g.kg^−1.^d^−1^, raising the question of whether daily protein intake above this level may further increase muscle mass. Results from several studies have also shown no further benefit to increases in muscle mass following CT with higher daily protein intakes [58,59,67,68]. Several interrelated factors may explain this finding, including older age and potentially associated anabolic resistance [58,59], insufficient RT volume [58,59], and inclusion of participants with obesity [67,68] that may specifically relate to a previously observed blunted MPS response to protein ingestion in such individuals [69]. Findings from the literature investigating changes in lean and FFM with different daily amounts of high protein availability following RT similarly show no added benefit. For instance, ingestion of 5.5 times the RDA of protein (4.4 g.kg^−1.^d^−1^) resulted in similar gains in FFM compared to 1.8 g.kg^−1.^d^−1^ in resistance-trained individuals who otherwise maintain the same training regimen [70]. Moreover, healthy-trained males and females consuming 3.4 g.kg^−1.^d^−1^ of protein showed no further increase in FFM compared to those ingesting 2.3 g.kg^−1.^d^−1^ [29]. However, a key difference between CT and RT performed in isolation is likely differences in inherent training volumes/loads. The greater training volume with CT can lead to situations of energy deficit and thus spending more time in a catabolic state, particularly if adequate nutritional practices are not enforced during periods of recovery and rest. While a recent meta-analysis reported the presence of an energy deficit to impair gains in lean mass with RT [71], findings from several studies show protein intake (in some cases up to three times the current RDA) can “rescue” declines in MPS with RT [72–74]. Thus, considering these findings and the aforementioned evidence demonstrating greater increases in muscle mass with CT with increased protein availability compared to levels around 1–1.1 g.kg^−1.^d^−1^ [30], it would be prudent for individuals participating in high-volume CT to consume protein intakes no less than 1.6 g.kg^−1.^d^−1^ with a likely upper limit of approximately 2.2–2.4 g.kg^−1.^d^−1^ to maximize increases in lean mass with CT. Total caloric energy must also be adequate in the context of CT to maximize increases in lean mass. While both higher protein groups in our current work reported significantly higher energy intakes compared to the lower protein groups, the similar increases in lean mass indicate caloric intake in these lower protein groups was still sufficient to support the cellular energy requirements to maximally facilitate muscle hypertrophy responses. In this regard, the caloric intake in the two lower protein groups (~122 kJ. kg^−1^.d^−1^, OR 29 kcal. kg^−1^.d^−1^, OR 2400 kcal.d^−1^, OR 10,053 kj.d^−1^) were comparable to energy intakes in previously reported studies demonstrating significant increases in muscle hypertrophy following CT [4,30], intuitively indicating this quantity of caloric intake can maximally promote increases in lean mass.

Among other body composition changes from our present work, we observed that BFP and VAT were reduced only in CT1 and remained unchanged in all other groups. This contrasts with our previous study in which fat mass remained unchanged with 12 weeks of CT while increasing in the RT group [4]. Possible explanations for the BFP loss in the CT1 group include elevated excess post-exercise oxygen consumption (EPOC) due to the combination of HIIT and MICT in the ET component [75–78] and lipolytic effects of epinephrine in response to HIIT previously identified [79] and subsequently expected to stimulate fat loss [80]. Notwithstanding, BFP remained unchanged in the CT2 group. Although there was no difference in training volume and modes between both CT groups, CT2 experienced a significant increase in energy intake during the intervention. Intuitively, it is possible that the high dietary protein intake in this group and potential for excess protein (i.e. beyond all tissue anabolic and metabolic needs) will eventually be converted to glucose (via gluconeogenesis) or ketone bodies and ultimately converted to glycogen or stored as fat [81,82].

### Aerobic capacity and performance measures

Another novel finding from this study was that increases in vertical jump performance were higher in the CT1 group compared to both RT groups. This was surprising given the attenuation in Wingate peak power with CT1 compared to RT2 previously discussed. Such discrepancy in these diverse power adaptation responses may relate to differences in neuromuscular activation between tests due to repetitive and high-force contractions of antagonistic muscles of the contralateral leg during a Wingate test compared to the more static and explosive nature of a single vertical jump [83]. Several studies have investigated vertical jump performance with CT and shown similar [50,84,85] or no post-training increase [86] compared to RT only. To our knowledge, this is the first study to investigate vertical jump performance with CT and high protein availability. Previous work following 8 weeks of RT showed no differences in vertical jump performance between daily protein intakes of either 2.3 g.kg^−1.^d^−1^ or 3.4 g.kg^−1.^d^−1^ in healthy trained males and females [29], indicating no beneficial effects of extra protein availability. We previously showed similar magnitude increases in the squat and countermovement jumps (which both closely resemble vertical jump performance) between 12 weeks of CT and RT in combination with a high protein diet (2 g.kg^−1.^d^−1^). Collectively, while it appears high dietary protein availability can confer beneficial effects on selective aspects of muscle power output (such as vertical jump), such positive responses need to be interpreted with caution, particularly considering muscle power adaptations may vary more depending on factors such as exercise order and recovery time between CT sessions [19].

Post-intervention increases in VO_2max_ were significantly higher with CT compared to RT in both dietary protein amounts. This is unsurprising given the aerobic training component of the CT program and supports findings from a meta-analysis of 21 studies that showed a significantly larger effect size for improvements in VO_2max_ with CT compared to RT only [87]. Previous work has demonstrated that the addition of RT to ET can increase endurance performance [88,89] and VO_2max_ [14,15,49,90–92], or at least produce similar magnitude increases in VO_2max_, compared to ET only [87,93,94]. Moreover, the capacity for CT to increase aerobic capacity does not appear to be dependent on exercise order within a CT program [64]. We recently demonstrated in a systematic review of 30 studies that protein ingestion following ET and/or HIIT significantly increases post-exercise MPS responses [95]. Interestingly, this capacity for exogenous protein intake to augment MPS responses with aerobic training and/or HIIT was mainly confined to mixed and myofibrillar proteins rather than mitochondrial proteins, which would appear counterintuitive based on a classical principle of training specificity [96]. Regardless, over extended periods of time (i.e. weeks, months), a recent systematic review and meta-analysis of 19 studies reported greater gains in O_2peak_ with protein supplementation compared to a control condition [97]. However, such findings are equivocal in the literature [98–100], and one study reported no further improvements in VO_2peak_ with 6 weeks CT between individuals consuming 3.5 g.kg^−1.^d^−1^ or 1.2 g.kg^−1.^d^−1^ of protein [7]. Nonetheless, considering the maximum number of pull-ups, another measure of (upper body) muscular endurance, was significantly greater with higher dietary protein availability (i.e. in CT2 and RT2 compared to CT1 and RT1) in our current work, we contend that available evidence overall supports the intake of adequate protein availability (i.e. at least 1.6 g.kg^−1.^d^−1^) to help mediate aerobic capacity and muscular endurance responses with CT.

### Biochemical markers of liver and kidney function

High protein diets have been associated with potential negative effects on kidney function, particularly glomerular filtration rate [101–103]. Specifically, it has been put forward that high and sustained dietary protein intake can ultimately lead to glomerular damage and eventual kidney damage and failure [104]. While such links may be more likely in individuals with compromised kidney function (such as chronic kidney disease) [105], the association between high dietary protein availability and reduced kidney and liver function appears much less evident in healthy individuals [106]. Considering the high protein components of our dietary intervention, which were two and four-fold higher than most national current RDAs for protein, we assessed several biochemical markers of kidney and liver function to determine whether 16 weeks of high protein availability alters these markers. Our results showed significant within-group increases in urea, creatinine, GGT, ALT, and AST post-intervention in both RT2 and CT2 groups consuming 3.2 g.kg^−1.^d^−1^ of protein, although no between-group differences compared with RT1 and CT1 groups were observed. Normal healthy ranges for these markers can vary depending on reference ranges used by local laboratories and an individual’s age. Compared to normal reference ranges, serum GGT, ALT, and AST values post-intervention in the RT2 and CT2 groups were still within normal reference ranges, although approaching the upper limits for these [107,108], while urea and creatinine levels were slightly above normal reference ranges [109,110]. While it is unknown whether these markers may continue to increase over longer periods of time (i.e. months to years), our results would indicate that individuals consuming very high protein diets (i.e. 3.2 g.kg^−1.^d^−1^) chronically may benefit from regular blood testing for continual monitoring of such markers to help confirm continual liver health.

### Limitations and conclusion

Limitations of our current work are acknowledged. Firstly, all HIIT and MICT sessions in our study design involved stationary cycling on an ergometer. As such, the capacity for overground and/or treadmill running to alter exercise adaptation responses with CT from our study design is unknown. This is an important factor considering the likelihood of greater exercise-induced muscle damage and/or slower recovery responses with running compared to cycling due to the eccentric and concentric contractile nature of running compared to concentric contractions only with cycling [111,112]. Moreover, running is a central exercise modality in many team sports, such as basketball and rugby that undergo CT programs for performance; thus, including this exercise modality in future CT studies can also provide new sports application knowledge. Another limitation of our work was no comparison to a non-exercise or placebo group (i.e. dietary protein intake at current RDA). Without these comparisons, it is difficult to completely elucidate the extent to which the exercise or dietary component contributed to increases in select adaptation responses observed.

In conclusion, we report that 16 weeks of RT and CT each performed 4 d. wk^−1^ in combination with high protein diets of either 1.6 or 3.2 g.kg^−1.^d^−1^results in similar improvements in muscle strength and mass, absolute upper body power, and performance adaptations. This suggests athletes can gain benefits when consuming 1.6 g.kg^−1^.d^−1^ to promote muscular adaptations from RT and CT, except for absolute lower body peak power. Moreover, due to increases in liver and kidney markers in CT2 and RT2 groups, a protein intake of 1.6 g.kg^−1.^d−NaN Invalid Date NaNbe safer long-term compared to greater amounts (i.e. 3.2 g.kg−1.d−1). Also, CT following 1.6 g.kg−1.d−1 resulted in similar improvements in vertical jump and pull-up in comparison to other groups; moreover, the increases in VO 2max were significantly greater than RT in isolation groups (both RT1 and RT2).

## Abbreviations

BFP: body fat percentage
FFM: fat-free mass
RT: resistance training
ET: endurance training
CT: concurrent training

## Supplementary Material

Supplemental Material

## Authors’ information

This was provided on the first page.

## Notes

**Datat availability statement** Data sharing is applicable.

## References

[1] Pourabbas, M, Bagheri, R, Hooshmand Moghadam, B, et al. Strategic ingestion of high-protein dairy milk during a resistance training program increases lean mass, strength, and power in trained young males. Nutrients. 2021;13(3):948. doi: 10.3390/nu13030948 33804259 PMC7999866

[2] Ashor, AW, Lara, J, Siervo, M, et al. Effects of exercise modalities on arterial stiffness and wave reflection: a systematic review and meta-analysis of randomized controlled trials. Plos One. 2014;9(10):e110034. doi: 10.1371/journal.pone.0110034 25333969 PMC4198209

[3] Hawley, JA. Adaptations of skeletal muscle to prolonged, intense endurance training. Clin Exp Pharmacol Physiol. 2002;29(3):218–610. doi: 10.1046/j.1440-1681.2002.03623.x 11906487

[4] Shamim, B, Devlin, BL, Timmins, RG, et al. Adaptations to concurrent training in combination with high protein availability: a comparative trial in healthy, recreationally active men. Sports Med. 2018;48(12):2869–2883. doi: 10.1007/s40279-018-0999-9 30341593 PMC6244626

[5] Davis, WJ, Wood, DT, Andrews, RG, et al. Concurrent training enhances athletes’ strength, muscle endurance, and other measures. J Strength Cond Res. 2008;22(5):1487–1502. doi: 10.1519/JSC.0b013e3181739f08 18714239

[6] Sabag, A, Najafi, A, Michael, S, et al. The compatibility of concurrent high intensity interval training and resistance training for muscular strength and hypertrophy: a systematic review and meta-analysis. J Sports Sci. 2018;36(21):2472–2483. doi: 10.1080/02640414.2018.1464636 29658408

[7] Forbes, SC, Bell, GJ. Whey protein isolate or concentrate combined with concurrent training does not augment performance, cardiorespiratory fitness, or strength adaptations. J Sports Med Phys Fitness. 2020;60(6):832–840. doi: 10.23736/S0022-4707.20.10314-1 32141277

[8] Baker, D. The effects of an in-season of concurrent training on the maintenance of maximal strength and power in professional and college-aged rugby league football players. J Strength Cond Res. 2001;15(2):172–177. doi: 10.1519/00124278-200105000-00004 11710401

[9] Argus, CK, Gill, N, Keogh, J, et al. Effects of a short-term pre-season training programme on the body composition and anaerobic performance of professional rugby union players. J Sports Sci. 2010;28(6):679–686. doi: 10.1080/02640411003645695 20397095

[10] Fyfe, JJ, Bishop, DJ, Stepto, NK. Interference between concurrent resistance and endurance exercise: molecular bases and the role of individual training variables. Sports Med. 2014;44(6):743–762. doi: 10.1007/s40279-014-0162-1 24728927

[11] Coffey, VG, Hawley, JA. Concurrent exercise training: do opposites distract? Journal Of Physiology. 2017;595(9):2883–2896. doi: 10.1113/JP272270 27506998 PMC5407958

[12] Craig, BW, Lucas, J, Pohlman, R, et al. The effects of running, weightlifting and a combination of both on growth hormone release. J Strength Cond Res. 1991;5(4):198–203. doi: 10.1519/00124278-199111000-00005

[13] Bell, G, Syrotuik, D, Martin, T, et al. Effect of concurrent strength and endurance training on skeletal muscle properties and hormone concentrations in humans. Eur J Appl Physiol. 2000;81(5):418–427. doi: 10.1007/s004210050063 10751104

[14] Häkkinen, K, Alen, M, Kraemer, W, et al. Neuromuscular adaptations during concurrent strength and endurance training versus strength training. Eur J Appl Physiol. 2003;89(1):42–52. doi: 10.1007/s00421-002-0751-9 12627304

[15] Fyfe, JJ, Bartlett, JD, Hanson, ED, et al. Endurance training intensity does not mediate interference to maximal lower-body strength gain during short-term concurrent training. Front Physiol. 2016;7:487. DOI:10.3389/fphys.2016.00487 27857692 PMC5093324

[16] Lundberg, TR, Fernandez-Gonzalo, R, Gustafsson, T, et al. Aerobic exercise does not compromise muscle hypertrophy response to short-term resistance training. J Appl Physiol. 2013;114(1):81–89. doi: 10.1152/japplphysiol.01013.2012 23104700

[17] Sale, D, Jacobs, I, MacDougall, J, et al. Comparison of two regimens of concurrent strength and endurance training. Med & Sci In Sports & Ex. 1990;22(3):348–356. doi: 10.1249/00005768-199006000-00012 2381303

[18] De Souza, E, Tricoli, V, Roschel, H, et al. Molecular adaptations to concurrent training. Int J Sports Med. 2013;34(3):207–213. doi: 10.1055/s-0032-1312627 23044732

[19] Schumann, M, Feuerbacher, JF, Sünkeler, M, et al. Compatibility of concurrent Aerobic and strength training for skeletal muscle size and function: an updated systematic review and meta-analysis. Sports Med 2021;52:1–12. 34757594 10.1007/s40279-021-01587-7 PMC8891239

[20] Khodadadi, F, Bagheri, R, Negaresh, R, et al. The effect of high-intensity interval training type on body fat percentage, fat and fat-free mass: a systematic review and meta-analysis of randomized clinical trials. J Clin Med. 2023;12(6):2291. doi: 10.3390/jcm12062291 36983289 PMC10054577

[21] Murach, KA, Bagley, JR. Skeletal muscle hypertrophy with concurrent exercise training: contrary evidence for an interference effect. Sports Med. 2016;46(8):1029–1039. doi: 10.1007/s40279-016-0496-y 26932769

[22] Bagheri, R, Moghadam, BH, Candow, DG, et al. Effects of Icelandic yogurt consumption and resistance training in healthy untrained older males. British Journal Of Nutrition. 2021;127(9):1334–1342. doi: 10.1017/S0007114521002166 34121642

[23] Morton, RW, Murphy, KT, McKellar, SR, et al. A systematic review, meta-analysis and meta-regression of the effect of protein supplementation on resistance training-induced gains in muscle mass and strength in healthy adults. Br J Sports Med. 2018;52(6):376–384. doi: 10.1136/bjsports-2017-097608 28698222 PMC5867436

[24] Campbell, WW. Synergistic use of higher-protein diets or nutritional supplements with resistance training to counter sarcopenia. Nutr Rev. 2007;65(9):416–422. doi: 10.1111/j.1753-4887.2007.tb00320.x 17958209

[25] Candow, DG, Chilibeck, PD, Facci, M, et al. Protein supplementation before and after resistance training in older men. Eur J Appl Physiol. 2006;97(5):548–556. doi: 10.1007/s00421-006-0223-8 16767436

[26] Bosse, JD, Dixon, BM. Dietary protein to maximize resistance training: a review and examination of protein spread and change theories. J Int Soc Sports Nutr. 2012;9(1):1–11. doi: 10.1186/1550-2783-9-42 22958314 PMC3518828

[27] Candow, DG, Burke, NC, Smith-Palmer, T, et al. Effect of whey and soy protein supplementation combined with resistance training in young adults. Int J Sport Nutr Exercise Metab. 2006;16(3):233–244. doi: 10.1123/ijsnem.16.3.233 16948480

[28] Rankin, JW, Goldman, LP, Puglisi, MJ, et al. Effect of post-exercise supplement consumption on adaptations to resistance training. J Am Coll Nutr. 2004;23(4):322–330. doi: 10.1080/07315724.2004.10719375 15310736

[29] Antonio, J, Ellerbroek, A, Silver, T, et al. A high protein diet (3.4 g/kg/d) combined with a heavy resistance training program improves body composition in healthy trained men and women–a follow-up investigation. J Int Soc Sports Nutr. 2015;12(1):1–9. doi: 10.1186/s12970-015-0100-0 26500462 PMC4617900

[30] Ormsbee, MJ, Willingham, BD, Marchant, T, et al. Protein supplementation during a 6-month concurrent training program: effect on body composition and muscular strength in sedentary individuals. Int J Sport Nutr Exercise Metab. 2018;28(6):619–628. doi: 10.1123/ijsnem.2018-0036 29485324

[31] Hartono, FA, Martin-Arrowsmith, PW, Peeters, WM, et al. The effects of dietary protein supplementation on acute changes in muscle protein synthesis and longer-term changes in muscle mass, strength, and aerobic capacity in response to concurrent resistance and endurance exercise in healthy adults: a systematic review. Sports Med. 2022;52:1–34. 10.1007/s40279-021-01620-9 35113389

[32] Camera, DM. Evaluating the effects of increased protein intake on muscle strength, hypertrophy and power adaptations with concurrent training: a narrative review. Sports Med. 2021;52(3):441–461. doi: 10.1007/s40279-021-01585-9 34822138

[33] Ghobadi, H, Attarzadeh Hosseini, SR, Rashidlamir, A, et al. Auto-regulatory progressive training compared to linear programming on muscular strength, endurance, and body composition in recreationally active males. Eur J Sport Sci. 2021;22(10):1543–1554. doi: 10.1080/17461391.2021.1963321 34346831

[34] Wilborn, CD, Taylor, LW, Outlaw, J, et al. The effects of pre-and post-exercise whey vs. casein protein consumption on body composition and performance measures in collegiate female athletes. J Sports Sci Med. 2013;12(1):74. 24149728 PMC3761774

[35] Cunha, PM, Nunes, JP, Tomeleri, CM, et al. Resistance training performed with single and multiple sets induces similar improvements in muscular strength, muscle mass, muscle quality, and IGF-1 in older women: A randomized controlled trial. J Strength Cond Res. 2020;34(4):1008–1016. doi: 10.1519/JSC.0000000000002847 30272625

[36] Colantonio, E, Barros, RV, MAPDM, K. Oxygen uptake during Wingate tests for arms and legs in swimmers and water polo players. Revista Brasileira de Medicina Do Esporte. 2003;9(3):141–144. doi: 10.1590/S1517-86922003000300003

[37] Ekblom‐Bak, E, Björkman, F, Hellenius, ML, et al. A new submaximal cycle ergometer test for prediction of VO2max. Scandinavian J Med Sci Sports. 2014;24(2):319–326. doi: 10.1111/sms.12014 23126417

[38] Björkman, F, Ekblom-Bak, E, Ekblom, Ö, et al. Validity of the revised Ekblom Bak cycle ergometer test in adults. Eur J Appl Physiol. 2016;116(9):1627–1638. doi: 10.1007/s00421-016-3412-0 27311582 PMC4983286

[39] Björkman, F, Eggers, A, Stenman, A, et al. Sex and maturity status affected the validity of a submaximal cycle test in adolescents. Acta Paediatrica. 2018;107(1):126–133. doi: 10.1111/apa.14080 28925577

[40] Spillane, M, Schwarz, N, Willoughby, DS. Heavy resistance training and peri-exercise ingestion of a multi-ingredient ergogenic nutritional supplement in males: effects on body composition, muscle performance and markers of muscle protein synthesis. J Sports Sci Med. 2014;13(4):894. 25435783 PMC4234960

[41] Haff, GG, Triplett, NT. Essentials of strength training and conditioning. 4th ed. Champaign, IL: Human kinetics; 2015.

[42] Vechin, FC, Conceição, MS, Telles, GD, et al. Interference phenomenon with concurrent strength and high-intensity interval training-based aerobic training: an updated model. Sports Med. 2021;51(4):599–605. doi: 10.1007/s40279-020-01421-6 33405189

[43] Antonio, J, Ellerbroek, A, Silver, T, et al. A high protein diet has no harmful effects: a one-year crossover study in resistance-trained males. J Nutr Metab. 2016;2016:1–5. DOI:10.1155/2016/9104792 PMC5078648 27807480

[44] Moore, DR, Robinson, MJ, Fry, JL, et al. Ingested protein dose response of muscle and albumin protein synthesis after resistance exercise in young men. Am J Clin Nutr. 2009;89(1):161–168. doi: 10.3945/ajcn.2008.26401 19056590

[45] Snijders, T, Res, PT, Smeets, JS, et al. Protein ingestion before sleep increases muscle mass and strength gains during prolonged resistance-type exercise training in healthy young men. J Nutr. 2015;145(6):1178–1184. doi: 10.3945/jn.114.208371 25926415

[46] Perez-Schindler, J, Hamilton, DL, Moore, DR, et al. Nutritional strategies to support concurrent training. Eur J Sport Sci. 2015;15(1):41–52. doi: 10.1080/17461391.2014.950345 25159707

[47] Hamilton, DL, Philp, A. Can AMPK mediated suppression of mTORC1 explain the concurrent training effect? Cell Mol Exerc Physiol. 2013;2(1):e4. doi: 10.7457/cmep.v2i1.e4

[48] Cohen, J. A power primer. Phychol Bulletin. 1992;112(1):155–159. doi: 10.1037/0033-2909.112.1.155 19565683

[49] Hickson, RC. Interference of strength development by simultaneously training for strength and endurance. Eur J Appl Physiol Occup Physiol. 1980;45(2–3):255–263. doi: 10.1007/BF00421333 7193134

[50] McCarthy, JP, Agre, JC, Graf, BK, et al. Compatibility of adaptive responses with combining strength and endurance training. Med & Sci In Sports & Ex. 1995;27(3):429–436. doi: 10.1249/00005768-199503000-00021 7752872

[51] McCarthy, JP, Pozniak, MA, Agre, JC. Neuromuscular adaptations to concurrent strength and endurance training. Medicine & Science In Sports & Exercise. 2002;34(3):511–519. doi: 10.1097/00005768-200203000-00019 11880817

[52] Petré, H, Hemmingsson, E, Rosdahl, H, et al. Development of maximal dynamic strength during concurrent resistance and endurance training in untrained, moderately trained, and trained individuals: a systematic review and meta-analysis. Sports Med. 2021;51(5):991–1010. doi: 10.1007/s40279-021-01426-9 33751469 PMC8053170

[53] De Souza, EO, Tricoli, V, Franchini, E, et al. Acute effect of two aerobic exercise modes on maximum strength and strength endurance. J Strength Cond Res. 2007;21(4):1286–1290. doi: 10.1519/00124278-200711000-00053 18076237

[54] Sporer, BC, Wenger, HA. Effects of aerobic exercise on strength performance following various periods of recovery. J Strength Cond Res. 2003;17(4):638–644. doi: 10.1519/00124278-200311000-00003 14636098

[55] Robineau, J, Babault, N, Piscione, J, et al. Specific training effects of concurrent aerobic and strength exercises depend on recovery duration. J Strength Cond Res. 2016;30(3):672–683. doi: 10.1519/JSC.0000000000000798 25546450

[56] Jäger, R, Kerksick, CM, Campbell, BI, et al. International society of sports nutrition position stand: protein and exercise. J Int Soc Sports Nutr. 2017;14(1):20. doi: 10.1186/s12970-017-0177-8 28642676 PMC5477153

[57] Walker, TB, Smith, J, Herrera, M, et al. The influence of 8 weeks of whey-protein and leucine supplementation on physical and cognitive performance. Int J Sport Nutr Exercise Metab. 2010;20(5):409–417. doi: 10.1123/ijsnem.20.5.409 20975109

[58] Ives, SJ, Norton, C, Miller, V, et al. Multi-modal exercise training and protein-pacing enhances physical performance adaptations independent of growth hormone and BDNF but may be dependent on IGF-1 in exercise-trained men. Growth Hormone IGF Res. 2017;32:60–70. DOI:10.1016/j.ghir.2016.10.002 27789212

[59] Arciero, PJ, Ives, SJ, Norton, C, et al. Protein-pacing and multi-component exercise training improves physical performance outcomes in exercise-trained women: the PRISE 3 study. Nutrients. 2016;8(6):332. doi: 10.3390/nu8060332 27258301 PMC4924173

[60] Tsitkanou, S, Spengos, K, Stasinaki, AN, et al. Effects of high‐intensity interval cycling performed after resistance training on muscle strength and hypertrophy. Scandinavian J Med Sci Sports. 2017;27(11):1317–1327. doi: 10.1111/sms.12751 27659479

[61] Linari, M, Bottinelli, R, Pellegrino, MA, et al. The mechanism of the force response to stretch in human skinned muscle fibres with different myosin isoforms. Journal Of Physiology. 2004;554(2):335–352. doi: 10.1113/jphysiol.2003.051748 14555725 PMC1664769

[62] Kazior, Z, Willis, SJ, Moberg, M, et al. Endurance exercise enhances the effect of strength training on muscle fiber size and protein expression of Akt and mTOR. Plos One. 2016;11(2):e0149082. doi: 10.1371/journal.pone.0149082 26885978 PMC4757413

[63] Eddens, L, van Someren, K, Howatson, G. The role of intra-session exercise sequence in the interference effect: a systematic review with meta-analysis. Sports Med. 2018;48(1):177–188. doi: 10.1007/s40279-017-0784-1 28917030 PMC5752732

[64] Murlasits, Z, Kneffel, Z, Thalib, L. The physiological effects of concurrent strength and endurance training sequence: A systematic review and meta-analysis. J Sports Sci. 2018;36(11):1212–1219. doi: 10.1080/02640414.2017.1364405 28783467

[65] Ballard, TL, Clapper, JA, Specker, BL, et al. Effect of protein supplementation during a 6-mo strength and conditioning program on insulin-like growth factor I and markers of bone turnover in young adults. Am J Clin Nutr. 2005;81(6):1442–1448. doi: 10.1093/ajcn/81.6.1442 15941900

[66] Longland, TM, Oikawa, SY, Mitchell, CJ, et al. Higher compared with lower dietary protein during an energy deficit combined with intense exercise promotes greater lean mass gain and fat mass loss: a randomized trial. Am J Clin Nutr. 2016;103(3):738–746. doi: 10.3945/ajcn.115.119339 26817506

[67] Cronin, O, Barton, W, Skuse, P, et al. A prospective metagenomic and metabolomic analysis of the impact of exercise and/or whey protein supplementation on the gut microbiome of sedentary adults. mSystems. 2018;3(3):e00044–18. doi: 10.1128/mSystems.00044-18 29719871 PMC5915698

[68] Weinheimer, EM, Conley, TB, Kobza, VM, et al. Whey protein supplementation does not affect exercise training-induced changes in body composition and indices of metabolic syndrome in middle-aged overweight and obese adults. J Nutr. 2012;142(8):1532–1539. doi: 10.3945/jn.111.153619 22718030 PMC3397339

[69] Beals, JW, Burd, NA, Moore, DR, et al. Obesity alters the muscle protein synthetic response to nutrition and exercise. Front Nutr. 2019;6:87. doi: 10.3389/fnut.2019.00087 31263701 PMC6584965

[70] Antonio, J, Peacock, CA, Ellerbroek, A, et al. The effects of consuming a high protein diet (4.4 g/kg/d) on body composition in resistance-trained individuals. J Int Soc Sports Nutr. 2014;11(1):19. doi: 10.1186/1550-2783-11-19 24834017 PMC4022420

[71] Murphy, C, Koehler, K. Energy deficiency impairs resistance training gains in lean mass but not strength: A meta‐analysis and meta‐regression. Scandinavian J Med Sci Sports. 2022;32(1):125–137. doi: 10.1111/sms.14075 34623696

[72] Areta, JL, Burke, LM, Camera, DM, et al. Reduced resting skeletal muscle protein synthesis is rescued by resistance exercise and protein ingestion following short-term energy deficit. Am J Physiol Endocrinol Metab. 2014;306(8):E989–E997. doi: 10.1152/ajpendo.00590.2013 24595305

[73] Hector, AJ, McGlory, C, Damas, F, et al. Pronounced energy restriction with elevated protein intake results in no change in proteolysis and reductions in skeletal muscle protein synthesis that are mitigated by resistance exercise. Faseb J. 2018;32(1):265–275. doi: 10.1096/fj.201700158RR 28899879

[74] Hudson, JL, Wang, Y, Bergia, RE III, et al. Protein intake greater than the RDA differentially influences whole-body lean mass responses to purposeful catabolic and anabolic stressors: a systematic review and meta-analysis. Adv Nutr. 2020;11(3):548–558. doi: 10.1093/advances/nmz106 31794597 PMC7231581

[75] Chan, HH, Burns, SF. Oxygen consumption, substrate oxidation, and blood pressure following sprint interval exercise. Appl Physiol Nutr Metab. 2013;38(2):182–187. doi: 10.1139/apnm-2012-0136 23438230

[76] Gore, C, Withers, R. The effect of exercise intensity and duration on the oxygen deficit and excess post-exercise oxygen consumption. Eur J Appl Physiol Occup Physiol. 1990;60(3):169–174. doi: 10.1007/BF00839153 2347316

[77] Hazell, TJ, Olver, TD, Hamilton, CD, et al. Two minutes of sprint-interval exercise elicits 24-hr oxygen consumption similar to that of 30 min of continuous endurance exercise. Int J Sport Nutr Exercise Metab. 2012;22(4):276–283. doi: 10.1123/ijsnem.22.4.276 22710610

[78] Laforgia, J, Withers, RT, Gore, CJ. Effects of exercise intensity and duration on the excess post-exercise oxygen consumption. J Sports Sci. 2006;24(12):1247–1264. doi: 10.1080/02640410600552064 17101527

[79] Zouhal, H, Jacob, C, Delamarche, P, et al. Catecholamines and the effects of exercise, training and gender. Sports Med. 2008;38(5):401–423. doi: 10.2165/00007256-200838050-00004 18416594

[80] Galster, AD, Clutter, WE, Cryer, PE, et al. Epinephrine plasma thresholds for lipolytic effects in man: measurements of fatty acid transport with [l-13C] palmitic acid. J Clin Investig. 1981;67(6):1729–1738. doi: 10.1172/JCI110211 7016921 PMC370750

[81] Hildebrandt, LA, Spennetta, T, Elson, C, et al. Utilization and preferred metabolic pathway of ketone bodies for lipid synthesis by isolated rat hepatoma cells. Am J Physiol Cell Physiol. 1995;269(1):C22–C7. doi: 10.1152/ajpcell.1995.269.1.C22 7631749

[82] Gannon, MC, Nuttall, FQ. Amino acid ingestion and glucose metabolism—a review. IUBMB Life. 2010;62(9):660–668. doi: 10.1002/iub.375 20882645

[83] Driss, T, Vandewalle, H. The measurement of maximal (anaerobic) power output on a cycle ergometer: a critical review. Bio Med Res Int. 2013;2013:1–40. doi: 10.1155/2013/589361 PMC3773392 24073413

[84] Mathieu, B, Robineau, J, Piscione, J, et al. Concurrent training programming: the acute effects of sprint interval exercise on the subsequent strength training. Sports. 2022;10(5):75. doi: 10.3390/sports10050075 35622484 PMC9145373

[85] Balabinis, CP, Psarakis, CH, Moukas, M, et al. Early phase changes by concurrent endurance and strength training. J Strength Cond Res. 2003;17(2):393–401. doi: 10.1519/1533-4287(2003)017<0393:EPCBCE>2.0.CO;2 12741884

[86] Glowacki, SP, Martin, SE, Maurer, A, et al. Effects of resistance, endurance, and concurrent exercise on training outcomes in men. Med & Sci In Sports & Ex. 2004;36:2119–2127. DOI:10.1249/01.MSS.0000147629.74832.52 15570149

[87] Wilson, JM, Marin, PJ, Rhea, MR, et al. Concurrent training: a meta-analysis examining interference of aerobic and resistance exercises. J Strength Cond Res. 2012;26(8):2293–2307. doi: 10.1519/JSC.0b013e31823a3e2d 22002517

[88] Aagaard, P, Andersen, JL. Effects of strength training on endurance capacity in top‐level endurance athletes. Scandinavian J Med Sci Sports. 2010;20:39–47. doi: 10.1111/j.1600-0838.2010.01197.x 20840561

[89] Rønnestad, BR, Hansen, EA, Raastad, T. Effect of heavy strength training on thigh muscle cross-sectional area, performance determinants, and performance in well-trained cyclists. Eur J Appl Physiol. 2010;108(5):965–975. doi: 10.1007/s00421-009-1307-z 19960350

[90] Mikkola, J, Rusko, H, Izquierdo, M, et al. Neuromuscular and cardiovascular adaptations during concurrent strength and endurance training in untrained men. Int J Sports Med. 2012;33(9):702–710. doi: 10.1055/s-0031-1295475 22706947

[91] Cadore, EL, Izquierdo, M, Pinto, SS, et al. Neuromuscular adaptations to concurrent training in the elderly: effects of intrasession exercise sequence. Age. 2013;35(3):891–903. doi: 10.1007/s11357-012-9405-y 22453934 PMC3636398

[92] Hickson, R, Dvorak, B, Gorostiaga, E, et al. Potential for strength and endurance training to amplify endurance performance. J Appl Physiol. 1988;65(5):2285–2290. doi: 10.1152/jappl.1988.65.5.2285 3209573

[93] Nelson, AG, Arnall, DA, Loy, SF, et al. Consequences of combining strength and endurance training regimens. Phys Ther. 1990;70(5):287–294. doi: 10.1093/ptj/70.5.287 2333326

[94] Psilander, N, Frank, P, Flockhart, M, et al. Adding strength to endurance training does not enhance aerobic capacity in cyclists. Scandinavian J Med Sci Sports. 2015;25(4):e353–e359. doi: 10.1111/sms.12338 25438613

[95] Bagheri, R, Robinson, I, Moradi, S, et al. Muscle protein synthesis responses following aerobic-based exercise or high-intensity interval training with or without protein ingestion: a systematic review. Sports Med. 2022;52(11):2713–2732. doi: 10.1007/s40279-022-01707-x 35675022 PMC9585015

[96] Coffey, VG, Hawley, JA. The molecular bases of training adaptation. Sports Med. 2007;37(9):737–763. doi: 10.2165/00007256-200737090-00001 17722947

[97] Lin, Y-N, Tseng, T-T, Knuiman, P, et al. Protein supplementation increases adaptations to endurance training: A systematic review and meta-analysis. Clin Nutr. 2021;40(5):3123–3132. doi: 10.1016/j.clnu.2020.12.012 33358231

[98] Forbes, SC, Bell, GJ. Whey protein isolate supplementation while endurance training does not Alter cycling performance or immune responses at rest or after exercise. Front Nutr. 2019;6:19. doi: 10.3389/fnut.2019.00019 30881958 PMC6406070

[99] Roberson, PA, Romero, MA, Mumford, PW, et al. Protein supplementation throughout 10 weeks of progressive run training is not beneficial for time trial improvement. Front Nutr. 2018;5:97. DOI:10.3389/fnut.2018.00097 30456213 PMC6230989

[100] Jonvik, KL, Paulussen, KJ, Danen, SL, et al. Protein supplementation does not augment adaptations to endurance exercise training. Med & Sci In Sports & Ex. 2019;51(10):2041. doi: 10.1249/MSS.0000000000002028 PMC6798744 31525168

[101] Ko, G-J, Rhee, CM, Kalantar-Zadeh, K, et al. The effects of high-protein diets on kidney health and longevity. J Am Soc Nephrol. 2020;31(8):1667–1679. doi: 10.1681/ASN.2020010028 32669325 PMC7460905

[102] Kalantar-Zadeh, K, Kramer, HM, Fouque, D. High-protein diet is bad for kidney health: unleashing the taboo. UK: Oxford University Press; 2020. 1–4. 10.1093/ndt/gfz216 31697325

[103] Schwingshackl, L, Hoffmann, G, Sands, JM. Comparison of high vs. normal/low protein diets on renal function in subjects without chronic kidney disease: a systematic review and meta-analysis. Plos One. 2014;9(5):e97656. doi: 10.1371/journal.pone.0097656 24852037 PMC4031217

[104] Brenner, BM, Meyer, TW, Hostetter, TH. Dietary protein intake and the progressive nature of kidney disease: the role of hemodynamically mediated glomerular injury in the pathogenesis of progressive glomerular sclerosis in aging, renal ablation, and intrinsic renal disease. N Engl J Med. 1982;307(11):652–659. doi: 10.1056/NEJM198209093071104 7050706

[105] Fouque, D, Laville, M, Boissel, JP 2006 . Low protein diets for chronic kidney disease in non diabetic adults. Cochrane Database Syst Rev. (2):1–10. 10.1002/14651858.CD001892.pub2 16625550

[106] Devries, MC, Sithamparapillai, A, Brimble, KS, et al. Changes in kidney function do not differ between healthy adults consuming higher-compared with lower-or normal-protein diets: a systematic review and meta-analysis. J Nutr. 2018;148(11):1760–1775. doi: 10.1093/jn/nxy197 30383278 PMC6236074

[107] Shivaraj, G, Prakash, D, Vinayak, H, et al. A review on laboratory liver function tests. Pan Afr Med J. 2009;3:17. 21532726 PMC2984286

[108] Nicoll, D, Detmer, W. Current medical diagnosis & treatment. Basic Principles of Diagnostic Test Use and Interpretation. 36th edn ed. Stamford, CT: Appleton & Lange; 1997. 1–16.

[109] Shahbaz, H, Gupta, M, Owais, A. Frequency, awareness, and symptoms of chikungunya among patients in a tertiary care hospital of Karachi: a cross-sectional study. Cureus. 2019;11(2). doi: 10.7759/cureus.4054 PMC6464461 31016081

[110] Hosten, AO. BUN and creatinine. Clinical methods: the history, physical, and laboratory examinations. 3rd ed. Salem, New Hampshire: Butterworths; 1990. 21250045

[111] Bijker, K, De Groot, G, Hollander, A. Differences in leg muscle activity during running and cycling in humans. Eur J Appl Physiol. 2002;87(6):556–561. doi: 10.1007/s00421-002-0663-8 12355196

[112] Peake, JM, Neubauer, O, Della Gatta, PA, et al. Muscle damage and inflammation during recovery from exercise. J Appl Physiol. 2017;122(3):559–570. doi: 10.1152/japplphysiol.00971.2016 28035017

