# Supplementary material for: Effects of 16 weeks of two different high-protein diets with either resistance or concurrent training on body composition, muscular strength and performance, and markers of liver and kidney function in resistance-trained males
Source: J Int Soc Sports Nutr. 2023 Jul 29;20(1):2236053. doi: 10.1080/15502783.2023.2236053 (PMC10388821; doi:10.1080/15502783.2023.2236053)
Supplement: Supplemental Material [file RSSN_A_2236053_SM7392.zip › Suppl/44.docx]

| **Supplementatry Table 1A.** Changes in body composition throughout the 16-week training intervention. | | | | | | | |
| --- | --- | --- | --- | --- | --- | --- | --- |
| **Measure** | **Time** | | | **ES** | **P** | | **η 2** |
|  | **Pre** | **Mid** | **Post** |  | **T** | **G × T** |  |
| Lean mass (kg) | | | | | | | |
| CT1 | 60.5 ± 6 | 61 ± 7 | 62.4 ± 6.3 ^a,b^ | 0.31 | p<0.001 | p=0.580 | 0.056 |
| CT2 | 59.7 ± 6.2 | 60.9 ± 6.1 | 61.8 ± 5.5 ^a^ | 0.38 |  |  |  |
| RT1 | 59.9 ± 6.6 | 61.4 ± 6.2 ^a^ | 61.9 ± 6.4 ^a^ | 0.32 |  |  |  |
| RT2 | 62.7 ± 9.2 | 63.4 ± 9.5 | 65.1 ± 9 ^a,b^ | 0.27 |  |  |  |
| EST. VAT mass (g) | | | | | | | |
| CT1 | 376.1 ± 221.5 | 347.6 ± 178.1 | 300.6 ± 157.9 ^a,b^ | -0.41 | p=0.022 | p=0.059 | 0.168 |
| CT2 | 323.7 ± 139.1 | 296.6 ± 111.6 | 310 ± 124.4 | -0.10 |  |  |  |
| RT1 | 330.6 ± 131 | 325.2 ± 138.3 | 326.7 ± 133.5 | -0.03 |  |  |  |
| RT2 | 313.6 ± 101.4 | 331.1 ± 109.2 | 304.4 ± 100.9 | -0.09 |  |  |  |
| BFP (%) | | | | | | | |
| CT1 | 21.8 ± 5.7 | 21.3 ± 5.1 | 19.8 ± 5.4 ^a^ | -0.36 | p=0.001 | p=0.218 | 0.098 |
| CT2 | 21.4 ± 4.1 | 20.1 ± 3.7 | 20.6 ± 3.3 | -0.20 |  |  |  |
| RT1 | 21.6 ± 3.4 | 20.5 ± 3.2 | 20.7 ± 3.8 | -0.26 |  |  |  |
| RT2 | 21 ± 4 | 20.6 ± 3.9 | 20.2 ± 3.5 | -0.23 |  |  |  |

^a^ p<0.05 different from pre ; ^b^ p<0.05 different from mid. **Abbreviations:** EST. VAT mass (g), Estimated visceral adipose tissue; BFP, body fat percentage; CT1, concurrent training + 1.6 g.kg^-1^.d^-1^; CT2, concurrent training + 3.2 g.kg^-1^.d^-1^; RT1, resistance training + 1.6 g.kg^-1^.d^-1^; RT2, resistance training + 3.2 g.kg^-1^.d^-1^; ES, effect size; η 2, group × time partial eta squared.

| **Supplementatry Table 1B.** The parameter estimates of group using Generalized Estimation Equation model. | | | | |
| --- | --- | --- | --- | --- |
| **Measure** | **Contrast** | **Mean difference (SE)** | **95% CI** | **p-value** |
| Lean mass (kg) | CT1 vs. CT2 | 0.44 (2.51) | -4.47 to 5.37 | 0.858 |
|  | CT1 vs. RT1 | 0.24 (2.60) | -4.86 to 5.34 | 0.926 |
|  | CT1 vs. RT2 | -2.44 (3.23) | -8.79 to 3.90 | 0.450 |
|  | CT2 vs. RT1 | -0.20 (2.49) | -5.10 to 4.68 | 0.934 |
|  | CT2 vs. RT2 | -2.89 (3.15) | -9.07 to 3.28 | 0.358 |
|  | RT1 vs. RT2 | -2.69 (3.22) | -9.01 to 3.63 | 0.404 |
| EST. VAT mass (g) | CT1 vs. CT2 | 31.33 (63.09) | -92.32 to 154.98 | 0.619 |
|  | CT1 vs. RT1 | 13.93 (64.64) | -112.75 to 140.63 | 0.829 |
|  | CT1 vs. RT2 | 25.06 (59.24) | -91.06 to 141.18 | 0.672 |
|  | CT2 vs. RT1 | -17.39 (52.32) | -119.95 to 85.16 | 0.740 |
|  | CT2 vs. RT2 | -6.27 (45.49) | -95.44 to 82.90 | 0.890 |
|  | RT1 vs. RT2 | 11.12 (47.62) | -82.22 to 104.47 | 0.815 |
| BFP (%) | CT1 vs. CT2 | 0.25 (1.86) | -3.40 to 3.91 | 0.891 |
|  | CT1 vs. RT1 | 0.05 (1.82) | -3.51 to 3.63 | 0.975 |
|  | CT1 vs. RT2 | 0.36 (1.86) | -3.29 to 4.02 | 0.844 |
|  | CT2 vs. RT1 | -0.19 (1.42) | -2.99 to 2.59 | 0.890 |
|  | CT2 vs. RT2 | 0.11 (1.48) | -2.79 to -3.01 | 0.940 |
|  | RT1 vs. RT2 | 0.30 (1.43) | -2.49 to 3.11 | 0.829 |

**Abbreviations:** EST. VAT mass (g), Estimated visceral adipose tissue; BFP, body fat percentage; CT1, concurrent training + 1.6 g.kg^-1^.d^-1^; CT2, concurrent training + 3.2 g.kg^-1^.d^-1^; RT1, resistance training + 1.6 g.kg^-1^.d^-1^; RT2, resistance training + 3.2 g.kg^-1^.d^-1^.
