# Supplementary material for: Effects of 16 weeks of two different high-protein diets with either resistance or concurrent training on body composition, muscular strength and performance, and markers of liver and kidney function in resistance-trained males
Source: J Int Soc Sports Nutr. 2023 Jul 29;20(1):2236053. doi: 10.1080/15502783.2023.2236053 (PMC10388821; doi:10.1080/15502783.2023.2236053)
Supplement: Supplemental Material [file RSSN_A_2236053_SM7392.zip › Suppl/55.docx]

| **Supplementary Table 2A.** Changes in performance throughout the 16-week training intervention. | | | | | | | |
| --- | --- | --- | --- | --- | --- | --- | --- |
| **Measure** | **Time** | | | **ES** | **P** | | **η^2^** |
|  | **Pre** | **Mid** | **Post** |  | **T** | **G × T** |  |
| Absolute chest press strength (kg) | | | | | | | |
| CT1 | 96.7 ± 21.5 | 99.2 ± 20.9 | 106.8 ± 20.7 ^a,b^ | 0.50 | p<0.001 | p=0.245 | 0.092 |
| CT2 | 100.8 ± 19.3 | 105.9 ± 21.2 ^a^ | 111.1 ± 20.9 ^a,b^ | 0.53 |  |  |  |
| RT1 | 98.2 ± 17.7 | 103 ± 17.3 ^a^ | 110.8 ± 16.4 ^a,b^ | 0.77 |  |  |  |
| RT2 | 107.8 ± 16.4 | 112.6 ± 16.3 ^a^ | 120.7 ± 17.4 ^a,b^ | 0.80 |  |  |  |
| Relative chest press strength (kg. kg BM^-1^) | | | | | | | |
| CT1 | 1.16 ± 0.24 | 1.18 ± 0.22 | 1.26 ± 0.23 ^a,b^ | 0.46 | p<0.001 | p=0.759 | 0.035 |
| CT2 | 1.25 ± 0.32 | 1.33 ± 0.37 ^a^ | 1.37 ± 0.35 ^a,b^ | 0.36 |  |  |  |
| RT1 | 1.22 ± 0.31 | 1.27 ± 0.30 ^a^ | 1.34 ± 0.29 ^a,b^ | 0.42 |  |  |  |
| RT2 | 1.28 ± 0.22 | 1.32 ± 0.26 | 1.40 ± 0.26 ^a,b^ | 0.55 |  |  |  |
| Chest press endurance (r) | | | | | | | |
| CT1 | 11.8 ± 2.4 | 13.2 ± 1.7 ^a^ | 12.6 ± 1.5 | 0.42 | p=0.020 | p=0.210 | 0.100 |
| CT2 | 13.4 ± 1.6 | 13.5 ± 1.8 | 12.3 ± 2.3 | -0.57 |  |  |  |
| RT1 | 11 ± 2.3 | 11.7 ± 2.2 | 11.9 ± 1.3 | 0.52 |  |  |  |
| RT2 | 12 ± 2 | 13.1 ± 0.9 ^a^ | 12.1 ± 1.6 ^b^ | 0.10 |  |  |  |
| Absolute leg press strength (kg) | | | | | | | |
| CT1 | 412.5 ± 76.7 | 438.3 ± 72.7 ^a^ | 485.1 ± 65.5 ^a,b^ | 1.07 | p<0.001 | p=0.973 | 0.009 |
| CT2 | 388.2 ± 73.5 | 413.6 ± 59.9 ^a^ | 463.1 ± 70.7 ^a,b^ | 1.08 |  |  |  |
| RT1 | 390.3 ± 70.8 | 422.8 ± 64.1 ^a^ | 472.7 ± 69.4 ^a,b^ | 1.23 |  |  |  |
| RT2 | 408.7 ± 69.5 | 436.9 ± 68.9 ^a^ | 485.5 ± 64.6 ^a,^^b^ | 1.20 |  |  |  |
| Relative leg press strength (kg. kg BM^-1^) | | | | | | | |
| CT1 | 4.97 ± 1.12 | 5.27 ± 1.03 ^a^ | 5.79 ± 0.98 ^a,b^ | 0.81 | p<0.001 | p=0.985 | 0.008 |
| CT2 | 4.85 ± 1.23 | 5.17 ± 1.14 ^a^ | 5.74 ± 1.32 ^a,b^ | 0.73 |  |  |  |
| RT1 | 4.83 ± 1.17 | 5.16 ± 1.04 ^a^ | 5.70 ± 1.13 ^a,b^ | 0.79 |  |  |  |
| RT2 | 4.87 ± 1.08 | 5.16 ± 1.11 ^a^ | 5.65 ± 0.96 ^a,b^ | 0.79 |  |  |  |
| Leg press endurance (r) | | | | | | | |
| CT1 | 14.1 ± 2.8 | 14.8 ± 2.8 | 14.7 ± 2.4 | 0.21 | p=0.022 | p=0.599 | 0.054 |
| CT2 | 15.3 ± 3.3 | 15.6 ± 3.4 ^a^ | 14.1 ± 3.2 | -0.37 |  |  |  |
| RT1 | 14.8 ± 3.1 | 14.9 ± 2.8 | 13.4 ± 1.9 | -0.56 |  |  |  |
| RT2 | 15.2 ± 2.2 | 15 ± 3.1 | 13.9 ± 2.9 | -0.55 |  |  |  |
| Absolute upper body power (w) | | | | | | | |
| CT1 | 469 ± 62.4 | 484.1 ± 58.9 ^a^ | 498.3 ± 58.1 ^a,b^ | 0.51 | p<0.001 | p=0.009 | 0.188 |
| CT2 | 505 ± 92.6 | 518.4 ± 96.2 ^a^ | 537 ± 92.9 ^a,b^ | 0.36 |  |  |  |
| RT1 | 456.9 ± 61.3 | 478 ± 62.9 ^a^ | 501 ± 63.7 ^a,b^ | 0.74 |  |  |  |
| RT2 | 509 ± 70.9 | 529.7 ± 74.7 ^a^ | 558.4 ± 72.3 ^a,b^ | 0.72 |  |  |  |
| Relative upper body power (watt. kg BM^-1^) | | | | | | | |
| CT1 | 5.63 ± 0.80 | 5.82 ± 0.81 ^a^ | 5.93 ± 0.81 ^a,b^ | 0.42 | p<0.001 | p=0.608 | 0.054 |
| CT2 | 6.32 ± 1.66 | 6.50 ± 1.82 ^a^ | 6.67 ± 1.73 ^a^ | 0.17 |  |  |  |
| RT1 | 5.63 ± 1.07 | 5.81 ± 0.99 ^a^ | 6.04 ± 1.08 ^a,b^ | 0.39 |  |  |  |
| RT2 | 6.05 ± 1.10 | 6.27 ± 1.32 ^a^ | 6.51 ± 1.30 ^a,b^ | 0.40 |  |  |  |
| Absolute lower body power (w) | | | | | | | |
| CT1 | 696.2 ± 91.8 | 708.2 ± 98.6 ^a^ | 732 ± 94.9 ^a,b^ | 0.40 | p<0.001 | p<0.001 | 0.348 |
| CT2 | 738.8 ± 83 | 749.5 ± 86.3 ^a^ | 780.7 ± 78.2 ^a,b^ | 0.54 |  |  |  |
| RT1 | 695.8 ± 53 | 733 ± 58.9 ^a^ | 764.8 ± 60.6 ^a,b^ | 1.27 |  |  |  |
| RT2 | 752.1 ± 68.8 | 785.3 ± 70.3 ^a^ | 817.8 ± 71.5 ^a,b^ | 0.98 |  |  |  |
| Relative lower body power (watt. kg BM^-1^) | | | | | | | |
| CT1 | 8.38 ± 1.36 | 8.52 ± 1.43 | 8.72 ± 1.37 ^a,b^ | 0.25 | p<0.001 | p=0.118 | 0.117 |
| CT2 | 9.21 ± 1.81 | 9.36 ± 1.96 ^a^ | 9.66 ± 1.83 ^a^ | 0.25 |  |  |  |
| RT1 | 8.60 ± 1.40 | 8.94 ± 1.36 ^a^ | 9.23 ± 1.39 ^a,b^ | 0.47 |  |  |  |
| RT2 | 8.96 ± 1.44 | 9.29 ± 1.59 ^a^ | 9.57 ± 1.62 ^a,b^ | 0.41 |  |  |  |
| Vertical jump (cm) | | | | | | | |
| CT1 | 50.6 ± 4.5 | 52 ± 3.8 ^a^ | 53.5 ± 4.5 ^a,b^ | 0.67 | p<0.001 | p=0.564 | 0.055 |
| CT2 | 47.8 ± 8.2 | 49.8 ± 7.8 ^a^ | 52.5 ± 6.7 ^a,b^ | 0.65 |  |  |  |
| RT1 | 43 ± 6.9 | 44.8 ± 5.8 ^a^ | 46.5 ± 6.4 ^a^ | 0.55 |  |  |  |
| RT2 | 44.9 ± 7.1 | 46.5 ± 6.8 ^a^ | 48.3 ± 5.8 ^a,b^ | 0.55 |  |  |  |
| Pull-up (r) | | | | | | | |
| CT1 | 11.3 ± 2.8 | 12.7 ± 1.6 | 14.9 ± 1.7 ^a,b^ | 1.60 | p<0.001 | p=0.172 | 0.109 |
| CT2 | 13.2 ± 3 | 15.1 ± 2.8 ^a^ | 17 ± 2.5 ^a,b^ | 1.43 |  |  |  |
| RT1 | 12.7 ± 2 | 15.6 ± 2 ^a^ | 17.4 ± 2.3 ^a,b^ | 2.23 |  |  |  |
| RT2 | 14.3 ± 4.3 | 16.9 ± 4.5 ^a^ | 19.3 ± 3.9 ^a,b^ | 1.27 |  |  |  |
| VO_2max_ (ml^-1^.kg^-1^.min^-1^) | | | | | | | |
| CT1 | 36 ± 4.5 | 42.5 ± 5.3 ^a^ | 46.3 ± 4.6 ^a,b^ | 2.33 | p<0.001 | p<0.001 | 0.749 |
| CT2 | 37.1 ± 6.4 | 43.1 ± 7.4 ^a^ | 45.4 ± 7.3 ^a,b^ | 1.25 |  |  |  |
| RT1 | 33.9 ± 5.4 | 34.1 ± 5.6 | 34.5 ± 4.9 | 0.12 |  |  |  |
| RT2 | 36.4 ± 6.9 | 37 ± 6.6 | 36.5 ± 7 | 0.01 |  |  |  |

^a^ p<0.05 different from pre ; ^b^ p<0.05 different from mid. **Abbreviations:** CT1, concurrent training + 1.6 g.kg^-1^.d^-1^; CT2, concurrent training + 3.2 g.kg^-1^.d^-1^; RT1, resistance training + 1.6 g.kg^-1^.d^-1^; RT2, resistance training + 3.2 g.kg^-1^.d^-1^; ES, effect size; η^2^, group × time partial eta squared.

| **Supplementary Table 2B.** The parameter estimates of group using Generalized Estimation Equation model. | | | | |
| --- | --- | --- | --- | --- |
| **Measure** | **Contrast** | **Mean difference (SE)** | **95% CI** | **p-value** |
| Absolute chest press strength (kg) | CT1vs. CT2 | -4.22 (8.35) | -20.61 to 12.15 | 0.613 |
|  | CT1vs. RT1 | -2.77 (7.76) | -17.99 to 12.44 | 0.721 |
|  | CT1vs. RT2 | -12.5 (7.72) | -27.64 to 2.64 | 0.106 |
|  | CT2 vs. RT1 | 1.45 (7.55) | -13.35 to 16.26 | 0.847 |
|  | CT2 vs. RT2 | -8.27 (7.51) | -23.00 to 6.45 | 0.271 |
|  | RT1 vs. RT2 | -9.72 (6.85) | -23.15 to 3.70 | 0.156 |
| Relative chest press strength (kg. kg BM^-1^) | CT1vs. CT2 | -0.10 (0.11) | -0.34 to 0.12 | 0.378 |
|  | CT1vs. RT1 | -0.07 (0.11) | -0.28 to 0.14 | 0.525 |
|  | CT1vs. RT2 | -0.13 (0.09) | -0.32 to 0.06 | 0.179 |
|  | CT2 vs. RT1 | 0.03 (0.13) | -0.22 to 0.29 | 0.786 |
|  | CT2 vs. RT2 | -0.02 (0.12) | -0.26 to 0.21 | 0.834 |
|  | RT1 vs. RT2 | -0.06 (0.11) | -0.27 to 0.15 | 0.584 |
| Chest press endurance (r) | CT1vs. CT2 | -0.60 (0.65) | -1.88 to 0.68 | 0.358 |
|  | CT1vs. RT1 | 0.92 (0.62) | -0.29 to 2.13 | 0.137 |
|  | CT1vs. RT2 | 0.12 (0.58) | -1.01 to 1.27 | 0.827 |
|  | CT2 vs. RT1 | 1.52 (0.63) | 0.27 to 2.77 | 0.017 |
|  | CT2 vs. RT2 | 0.72 (0.60) | -0.45 to 1.91 | 0.226 |
|  | RT1 vs. RT2 | -0.79 (0.56) | -1.90 to 0.31 | 0.159 |
| Absolute leg press strength (kg) | CT1vs. CT2 | 23.22 (28.05) | -31.75 to 78.21 | 0.408 |
|  | CT1vs. RT1 | 17.26 (27.52) | -36.67 to 71.20 | 0.530 |
|  | CT1vs. RT2 | 1.71 (26.75) | -50.71 to 54.15 | 0.949 |
|  | CT2 vs. RT1 | -5.96 (28.320 | -61.47 to 49.55 | 0.833 |
|  | CT2 vs. RT2 | -21.50 (27.57) | -75.55 to 32.54 | 0.435 |
|  | RT1 vs. RT2 | -15.54 (27.03) | -68.53 to 37.43 | 0.565 |
| Relative leg press strength (kg. kg BM^-1^) | CT1vs. CT2 | 0.08 (0.46) | -0.83 to 0.99 | 0.861 |
|  | CT1vs. RT1 | 0.11 (0.43) | -0.74 to 0.97 | 0.799 |
|  | CT1vs. RT2 | 0.11 (0.40) | -0.68 to 0.92 | 0.771 |
|  | CT2 vs. RT1 | 0.03 (0.49) | -0.93 to 0.99 | 0.951 |
|  | CT2 vs. RT2 | 0.03 (0.46) | -0.87 to 0.94 | 0.935 |
|  | RT1 vs. RT2 | 0.007 (0.43) | -0.85 to 0.86 | 0.986 |
| Leg press endurance (r) | CT1vs. CT2 | -0.38 (1.09) | -2.52 to 1.75 | 0.725 |
|  | CT1vs. RT1 | 0.26 (0.91) | -1.52 to 2.05 | 0.772 |
|  | CT1vs. RT2 | -0.15 (0.85) | -1.83 to 1.52 | 0.857 |
|  | CT2 vs. RT1 | 0.64 (1.09) | -1.50 to 2.79 | 0.555 |
|  | CT2 vs. RT2 | 0.23 (1.05) | -1.83 to 2.29 | 0.827 |
|  | RT1 vs. RT2 | -0.41 (0.86) | -2.11 to 1.27 | 0.628 |
| Absolute upper body power (w) | CT1vs. CT2 | -37.36 (31.73) | -99.56 to 24.83 | 0.239 |
|  | CT1vs. RT1 | 4.68 (24.81) | -43.95 to 53.31 | 0.850 |
|  | CT1vs. RT2 | -50.04 (26.77) | -102.52 to 2.43 | 0.062 |
|  | CT2 vs. RT1 | 42.04 (32.05) | -20.78 to 104.87 | 0.190 |
|  | CT2 vs. RT2 | -12.68 (33.59) | -78.52 to 53.16 | 0.706 |
|  | RT1 vs. RT2 | -54.72 (27.15) | -107.94 to -1.51 | 0.044 |
| Relative upper body power (watt. kg BM^-1^) | CT1vs. CT2 | -0.71 (0.53) | -1.77 to 0.34 | 0.185 |
|  | CT1vs. RT1 | -0.05 (0.38) | -0.80 to 0.69 | 0.886 |
|  | CT1vs. RT2 | -0.51 (0.41) | -1.32 to 0.30 | 0.216 |
|  | CT2 vs. RT1 | 0.66 (0.57) | -0.47 to 1.79 | 0.252 |
|  | CT2 vs. RT2 | 0.20 (0.59) | -0.96 to 1.37 | 0.736 |
|  | RT1 vs. RT2 | -0.45 (0.46) | -1.36 to 0.44 | 0.321 |
| Absolute lower body power (w) | CT1vs. CT2 | -45.59 (35.34) | -114.86 to 23.67 | 0.197 |
|  | CT1vs. RT1 | -16.13 (31.24) | -77.37 to 45.10 | 0.606 |
|  | CT1vs. RT2 | -70.81 (33.42) | -136.32 to -5.31 | 0.034 |
|  | CT2 vs. RT1 | 29.45 (28.16) | -25.74 to 84.65 | 0.296 |
|  | CT2 vs. RT2 | -25.22 (30.55) | -85.11 to 34.66 | 0.409 |
|  | RT1 vs. RT2 | -54.68 (25.70) | -105.06 to -4.29 | 0.033 |
| Relative lower body power (watt. kg BM^-1^) | CT1vs. CT2 | -0.88 (0.65) | -2.16 to 0.39 | 0.175 |
|  | CT1vs. RT1 | -0.36 (0.56) | -1.46 to 0.73 | 0.517 |
|  | CT1vs. RT2 | -0.71 (0.58) | -1.86 to 0.43 | 0.223 |
|  | CT2 vs. RT1 | 0.52 (0.65) | -0.76 to 1.81 | 0.427 |
|  | CT2 vs. RT2 | 0.17 (0.68) | -1.16 to 1.50 | 0.802 |
|  | RT1 vs. RT2 | -0.35 (0.59) | -1.51 to 0.81 | 0.553 |
| Vertical jump (cm) | CT1vs. CT2 | 1.91 (2.46) | -2.92 to 6.74 | 0.438 |
|  | CT1vs. RT1 | 7.31 (2.26) | 2.88 to 11.74 | 0.001 |
|  | CT1vs. RT2 | 5.45 (2.22) | 1.09 to 9.81 | 0.014 |
|  | CT2 vs. RT1 | 5.40 (2.85) | -0.18 to 10.99 | 0.058 |
|  | CT2 vs. RT2 | 3.54 (2.82) | -1.99 to 9.07 | 0.210 |
|  | RT1 vs. RT2 | -5.40 (2.85) | -10.99 to 0.18 | 0.058 |
| Pull up (r) | CT1vs. CT2 | -2.05 (0.98) | -3.98 to -0.13 | 0.036 |
|  | CT1vs. RT1 | -1.98 (0.83) | -3.62 to -0.34 | 0.018 |
|  | CT1vs. RT2 | -3.74 (1.29) | -6.27 to -1.20 | 0.004 |
|  | CT2 vs. RT1 | -0.07 (0.98) | -1.84 to 1.99 | 0.941 |
|  | CT2 vs. RT2 | -1.68 (1.38) | -4.40 to 1.04 | 0.226 |
|  | RT1 vs. RT2 | -1.75 (1.29) | -4.28 to 0.77 | 0.174 |
| VO_2max_ (ml^-1^.kg^-1^.min^-1^) | CT1vs. CT2 | -0.09 (2.32) | -4.65 to 4.46 | 0.968 |
|  | CT1vs. RT1 | 7.00 (1.94) | 3.19 to 10.81 | <0.001 |
|  | CT1vs. RT2 | 4.72 (2.35) | 0.10 to 9.34 | 0.045 |
|  | CT2 vs. RT1 | 7.09 (2.44) | 2.30 to 11.88 | 0.004 |
|  | CT2 vs. RT2 | 4.82 (2.78) | -0.63 to 10.28 | 0.083 |
|  | RT1 vs. RT2 | -2.27 (2.47) | -7.12 to 2.57 | 0.358 |

**Abbreviations:** CT1, concurrent training + 1.6 g.kg^-1^.d^-1^; CT2, concurrent training + 3.2 g.kg^-1^.d^-1^; RT1, resistance training + 1.6 g.kg^-1^.d^-1^; RT2, resistance training + 3.2 g.kg^-1^.d^-1^.
