# Supplementary material for: Effects of 16 weeks of two different high-protein diets with either resistance or concurrent training on body composition, muscular strength and performance, and markers of liver and kidney function in resistance-trained males
Source: J Int Soc Sports Nutr. 2023 Jul 29;20(1):2236053. doi: 10.1080/15502783.2023.2236053 (PMC10388821; doi:10.1080/15502783.2023.2236053)
Supplement: Supplemental Material [file RSSN_A_2236053_SM7392.zip › Suppl/66.docx]

| **Supplementary Table 3A.** Changes in biochemical markers throughout the 16-week training intervention. | | | | | | | |
| --- | --- | --- | --- | --- | --- | --- | --- |
| **Measure** | **Time** | | | **ES** | **P** | | **η^2^** |
|  | **Pre** | **Mid** | **Post** |  | **T** | **G × T** |  |
| GGT (u/l) | | | | | | | |
| CT1 | 30.1 ± 5.4 | 31.4 ± 6.1 | 31.9 ± 5.5 | 0.33 | p<0.001 | p=0.107 | 0.126 |
| CT2 | 29.2 ± 6.2 | 35.9 ± 5.5 | 39.3 ± 4.4 | 1.98 |  |  |  |
| RT1 | 30.8 ± 4.7 | 32.1 ± 5.1 | 35.2 ± 5.8 | 0.88 |  |  |  |
| RT2 | 31.1 ± 6.7 | 35 ± 6.3 | 38.1 ± 8.5 | 0.96 |  |  |  |
| AST (u/l) | | | | | | | |
| CT1 | 29.6 ± 7.6 | 31.9 ± 5.9 | 34.4 ± 5.4 | 0.76 | p<0.001 | p=0.479 | 0.065 |
| CT2 | 27.1 ± 5.7 | 32.1 ± 7.1 | 37.9 ± 4 | 2.32 |  |  |  |
| RT1 | 25.7 ± 6.8 | 29.3 ± 6.2 | 30.6 ± 3.6 | 0.97 |  |  |  |
| RT2 | 28.8 ± 8.1 | 31 ± 7.4 | 37.1 ± 7.3 | 1.12 |  |  |  |
| ALT (u/l) | | | | | | | |
| CT1 | 24.1 ± 5.9 | 27.4 ± 5.4 | 30.6 ± 6.8 | 1.07 | p<0.001 | p=0.989 | 0.009 |
| CT2 | 27.9 ± 6.6 | 32.1 ± 5.1 | 36.1 ± 5.8 | 1.37 |  |  |  |
| RT1 | 26.1 ± 6.5 | 29 ± 5.5 | 31.9 ± 7.6 | 0.86 |  |  |  |
| RT2 | 29.1 ± 7.7 | 31.5 ± 5.3 | 36.1 ± 6.9 | 0.99 |  |  |  |
| Urea (mg/dl) | | | | | | | |
| CT1 | 17.9 ± 5.2 | 15.2 ± 4.5 | 19.1 ± 5.2 | 0.25 | p=0.003 | p=0.520 | 0.061 |
| CT2 | 16 ± 6 | 17.1 ± 4.1 | 20.4 ± 3.2 | 1 |  |  |  |
| RT1 | 17 ± 4.1 | 18 ± 5.2 | 18.4 ± 4.3 | 0.36 |  |  |  |
| RT2 | 18 ± 5.3 | 18.8 ± 4 | 22.8 ± 3.7 | 1.12 |  |  |  |
| Creatinine (mg/dl) | | | | | | | |
| CT1 | 1.11 ± 0.18 | 1.24 ± 0.22 | 1.32 ± 0.24 | 0.98 | p<0.001 | p=0.947 | 0.020 |
| CT2 | 1.06 ± 0.15 | 1.28 ± 0.16 | 1.33 ± 0.22 | 1.5 |  |  |  |
| RT1 | 1.18 ± 0.23 | 1.25 ± 0.22 | 1.34 ± 0.17 | 0.85 |  |  |  |
| RT2 | 1.15 ± 0.18 | 1.30 ± 0.17 | 1.38 ± 0.23 | 1.13 |  |  |  |

^a^ p<0.05 different from pre ; ^b^ p<0.05 different from mid. **Abbreviations.** GGT, Gamma-glutamyl transferase; AST, Aspartate transaminase; ALT, Alanine transaminase; CT1, concurrent training + 1.6 g.kg^-1^.d^-1^; CT2, concurrent training + 3.2 g.kg^-1^.d^-1^; RT1, resistance training + 1.6 g.kg^-1^.d^-1^; RT2, resistance training + 3.2 g.kg^-1^.d^-1^; ES, effect size; η^2^, group × time partial eta squared.

| **Supplementary Table 3B.** The parameter estimates of group using Generalized Estimation Equation model. | | | | |
| --- | --- | --- | --- | --- |
| **Measure** | **Contrast** | **Mean difference (SE)** | **95% CI** | **p-value** |
| GGT (u/l) | CT1vs. CT2 | -3.48 (1.92) | -7.25 to 0.28 | 0.070 |
|  | CT1vs. RT1 | -1.77 (1.70) | -5.10 to 1.56 | 0.297 |
|  | CT1vs. RT2 | -3.55 (2.30) | -8.06 to 0.97 | 0.124 |
|  | CT2 vs. RT1 | 1.71 (1.35) | -0.94 to 4.37 | 0.207 |
|  | CT2 vs. RT2 | -0.06 (2.06) | -4.10 to 3.98 | 0.976 |
|  | RT1 vs. RT2 | -1.77 (1.85) | -5.42 to 1.87 | 0.340 |
| AST (u/l) | CT1vs. CT2 | -0.41 (1.42) | -3.20 to 2.37 | 0.772 |
|  | CT1vs. RT1 | 3.55 (1.63) | 0.35 to 6.76 | 0.030 |
|  | CT1vs. RT2 | -0.48 (2.12) | -4.65 to 3.69 | 0.822 |
|  | CT2 vs. RT1 | 3.96 (1.38) | 1.24 to 6.68 | 0.004 |
|  | CT2 vs. RT2 | -0.70 (1.94) | -3.88 to 3.74 | 0.972 |
|  | RT1 vs. RT2 | -4.03 (2.10) | -8.16 to 0.10 | 0.056 |
| ALT (u/l) | CT1vs. CT2 | -4.61 (1.38) | -7.32 to -1.90 | 0.001 |
|  | CT1vs. RT1 | -1.56 (1.37) | -4.24 to 1.12 | 0.255 |
|  | CT1vs. RT2 | -4.92 (2.07) | -8.98 to -0.87 | 0.017 |
|  | CT2 vs. RT1 | 3.05 (1.18) | 0.73 to 5.38 | 0.010 |
|  | CT2 vs. RT2 | -0.31 (1.95) | -4.14 to 3.52 | 0.873 |
|  | RT1 vs. RT2 | -3.36 (1.94) | -7.18 to 0.45 | 0.084 |
| Urea (mg/dl) | CT1vs. CT2 | -0.38 (1.16) | -2.66 to 1.89 | 0.742 |
|  | CT1vs. RT1 | -0.35 (1.08) | -2.48 to 1.79 | 0.751 |
|  | CT1vs. RT2 | -2.42 (1.27) | -4.92 to 0.09 | 0.059 |
|  | CT2 vs. RT1 | 0.04 (1.03) | -1.98 to 2.06 | 0.971 |
|  | CT2 vs. RT2 | -2.03 (1.23) | -4.44 to 0.38 | 0.098 |
|  | RT1 vs. RT2 | -2.07 (1.16) | -4.34 to 0.20 | 0.074 |
| Creatinine (mg/dl) | CT1vs. CT2 | 0.0002 (0.04) | -0.09 to 0.09 | 0.997 |
|  | CT1vs. RT1 | -0.03 (0.03) | -0.11 to 0.04 | 0.401 |
|  | CT1vs. RT2 | -0.50 (0.05) | -0.16 to 0.05 | 0.325 |
|  | CT2 vs. RT1 | -0.03 (0.04) | -0.12 to 0.05 | 0.455 |
|  | CT2 vs. RT2 | -0.05 (0.05) | -0.16 to 0.06 | 0.356 |
|  | RT1 vs. RT2 | -0.02 (0.05) | -0.12 to 0.08 | 0.685 |

**Abbreviations.** GGT, Gamma-glutamyl transferase; AST, Aspartate transaminase; ALT, Alanine transaminase; CT1, concurrent training + 1.6 g.kg^-1^.d^-1^; CT2, concurrent training + 3.2 g.kg^-1^.d^-1^; RT1, resistance training + 1.6 g.kg^-1^.d^-1^; RT2, resistance training + 3.2 g.kg^-1^.d^-1^.
